# Macromolecular composition of phloem exudate from white lupin (*Lupinus albus *L.)

**DOI:** 10.1186/1471-2229-11-36

**Published:** 2011-02-22

**Authors:** Caren Rodriguez-Medina, Craig A Atkins, Anthea J Mann, Megan E Jordan, Penelope MC Smith

**Affiliations:** 1 INRA Center Colmar. France; 2 School of Plant Biology, The University of Western Australia, Crawley. WA 6009. Australia; 3 School of Biological Science, The University of Sydney. NSW 2006. Australia

## Abstract

**Background:**

Members of the legume genus *Lupinus *exude phloem 'spontaneously' from incisions made to the vasculature. This feature was exploited to document macromolecules present in exudate of white lupin (*Lupinus albus *[L.] *cv *Kiev mutant), in particular to identify proteins and RNA molecules, including microRNA (miRNA).

**Results:**

Proteomic analysis tentatively identified 86 proteins from 130 spots collected from 2D gels analysed by partial amino acid sequence determination using MS/MS. Analysis of a cDNA library constructed from exudate identified 609 unique transcripts. Both proteins and transcripts were classified into functional groups. The largest group of proteins comprised those involved in metabolism (24%), followed by protein modification/turnover (9%), redox regulation (8%), cell structural components (6%), stress and defence response (6%) with fewer in other groups. More prominent proteins were cyclophilin, ubiquitin, a glycine-rich RNA-binding protein, a group of proteins that comprise a glutathione/ascorbate-based mechanism to scavenge oxygen radicals, enzymes of glycolysis and other metabolism including methionine and ethylene synthesis. Potential signalling macromolecules such as transcripts encoding proteins mediating calcium level and the Flowering locus T (FT) protein were also identified. From around 330 small RNA clones (18-25 nt) 12 were identified as probable miRNAs by homology with those from other species. miRNA composition of exudate varied with site of collection (e.g. upward versus downward translocation streams) and nutrition (e.g. phosphorus level).

**Conclusions:**

This is the first inventory of macromolecule composition of phloem exudate from a species in the Fabaceae, providing a basis to identify systemic signalling macromolecules with potential roles in regulating development, growth and stress response of legumes.

## Background

Vascular plants have a well developed translocation system that facilitates transport of nutrients and particularly photoassimilates between organs. This vascular system is comprised of phloem and xylem conducting elements. The phloem vascular tissue in angiosperms is comprised of arrays of sieve element (SE)/companion cell (CC) complexes [1]. During their differentiation, the SE undergoes a selective autophagy which results in breakdown of the nucleus and tonoplast along with loss of ribosomes, Golgi and microtubules. Consequently, mature SE exhibit mostly a thin layer of parietal cytoplasm with stacked endoplasmic reticulum, some plastids and a small number of dilated mitochondria [2]. It is generally believed that the enucleate SE has lost the capacity for protein synthesis and has limited metabolic activity. CC must then participate in the maintenance and functioning of the enucleate SE [3]. Adjacent SE and CC are connected through branched plasmodesmata responsible for the exchange of small solutes and macromolecules in the SE/CC complex [1]. Thus, macromolecules identified in the mature SE are assumed to have been synthesized in and imported from an associated CC through plasmodesmatal connection [4].

Proteomic analyses of phloem exudates collected from incisions to the vasculature of a number of species that either 'bleed' spontaneously (e.g. castor bean [5], cucurbits [6,7] and *Brassica napus *[8]), or, in which exudation is aided by application of a chelator have shown a broad range of proteins, a small number of which are common with those identified in phloem exudate collected by stylectomy [9]. While together these data indicate that the phloem stream contains many proteins, it is not clear which of these are translocated and, more importantly, which have a function dependent on their long distance transport.

Numerous transcripts have been identified in phloem exudates collected not only from incisions to the vasculature in *Arabidopsis *[10], melon [11], and castor bean [12] but also by stylectomy from rice [13] and barley [14,15]. The presence of transcripts in phloem exudate suggests the concept of an RNA-based signalling network that functions in the control of plant development [16]. However, there are few transcripts for which translocation has been demonstrated and the need for translocation established [17-20].

Functional analysis of proteins and transcripts identified in phloem exudates revealed a wide range of processes including metabolism, responses to stress, transport, detoxification of reactive oxygen species (ROS), DNA/RNA binding, signalling and protein turnover. Recent studies have also revealed the presence of small RNA molecules, including microRNAs (miRNAs), in phloem exudates from cucurbits [21], *Brassica napus *[22], and *Malus domestica *(apple) [23]. There is a growing body of evidence linking miRNAs to the regulation of nutritional balance in plants and particularly to changes in P_i _and N status [24-26] and to S uptake [27]. These nutrients are translocated and distributed in organs as a consequence of 'source- sink interactions' raising the possibility that translocated miRNAs are involved in regulating these interactions.

While proteomic and RNA analysis of phloem exudates has been applied successfully to a number of dicotyledon species and, with the aid of sap sucking insects, to rice, barley, and apple, similar detailed analyses of proteins and RNA in exudates from legume species have been lacking. The ability to collect phloem exudates, both readily and in substantial volume from white lupin, without the use of a chelating agent, provides a valuable tool for studying the macromolecular composition of such exudates in a legume. In this study, partial sequence determination by MS/MS and subsequent protein database searches, were used to tentatively identify proteins separated using 2D gel electrophoresis from lupin phloem exudate collected mainly from developing fruits and the inflorescence raceme. These exudates were also analysed for RNA species including transcripts and miRNAs.

This is the first study to provide information on macromolecules present in the phloem exudate of a member of the Fabaceae. The information obtained adds further insights into the properties of the SE/CC complex and provides a basis for future studies seeking to identify potential systemic signals that may play a role in a communication network trafficking information around the plant, regulating specific developmental processes and responding to environmental cues.

## Results

### Lupin phloem exudates contain many proteins

**Figure 1:**
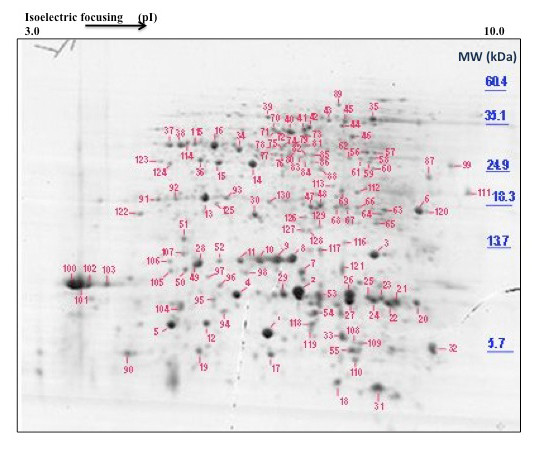
**Typical 2D gel electrophoresis separation of polypeptides in *L. albus *phloem exudate**. Phloem exudate was collected from the vasculature of developing fruits and the inflorescence raceme. 1 mg of protein was separated and stained using colloidal Coomassie Brilliant Blue G250. Protein spots were excised from the gel, digested with trypsin and analysed by partial sequence determination by MS/MS and subsequently identified using database searches. The positions of molecular mass markers are shown to the right of the figure and the pH gradient is indicated at the top of the gel.

**Figure 2:**
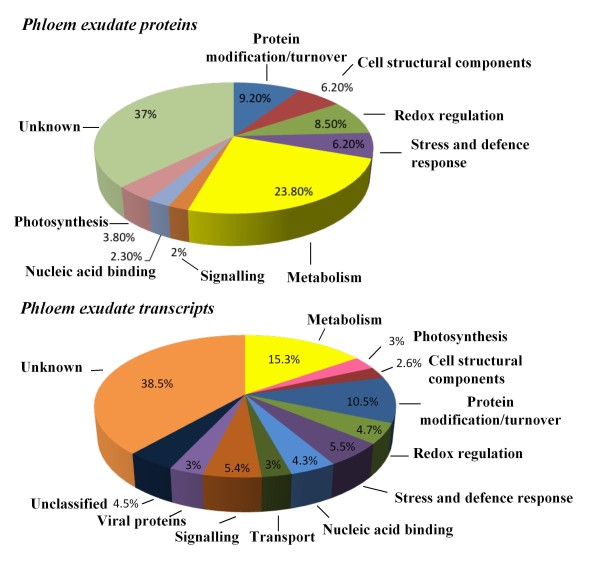
**Functional categorisation of proteins and transcripts identified in *L. albus *phloem exudate**. Phloem exudate was collected from pod sutures and inflorescence raceme by the incision method.

Separation of proteins on 2D gels permitted resolution of more than 200 Coomassie-staining spots (Figure 1). Of these, 130 were collected and partially sequenced by MS/MS. Representative spectra for a number of spots are shown in Additional file 1. Proteins from 52 spots were tentatively identified by the high level of identity of two or more peptides to sequences in current databases (protein or EST). For many spots an exact match to a deduced protein from a lupin EST was made. An additional 34 spots had single peptide matches to a known protein or a protein encoded by a lupin EST. These identifications were treated with caution but are included here to show the possible components of phloem. Additional file 2 shows the full list of identified proteins as well as the partial amino acid sequences used for identification and the BLAST search results. The 86 proteins with peptide matches corresponded to 55 unique accession numbers as some of the identified proteins were present in more than one spot. Of the sequenced proteins, 37% were classified as 'unknown' (Figure 2). This group included nine spots that contained peptides either at too low concentration or that exhibited adverse fragmentation behaviour resulting in poor spectra that were difficult to interpret, and 38 spots showing no significant homology to any protein in the database or that matched proteins of an unknown function. Some of the more prominent protein spots (4, 8, 9, 10, 11, 13 and 16 in Figure 1) were in this latter category. The prominent proteins that were identified included cyclophilin (spots 100 and 101), a glycine-rich RNA-binding protein (spots 26 and 27), and a cysteine proteinase inhibitor (spot 1).

### A large number of transcripts are present in lupin phloem exudate

A total of 1063 clones were sequenced from a cDNA library constructed from mRNA isolated from phloem exudate. Of these sequences, 192 were excluded due to low quality of the sequence and 144 ESTs did not show significant similarity to any sequence in the databases searched. A total of 609 unique transcripts corresponding to 727 ESTs were identified (Genbank accession numbers GW583301 to GW583999). 73% of all ESTs were singletons. 176 redundant EST sequences were assembled into 67 contigs with an average of 2.6 ESTs per contig. Additional file 3 shows the full list of sequenced clones as well as the BLAST search results.

**Figure 3:**
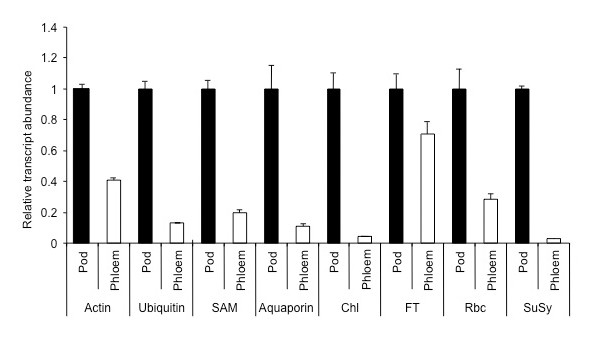
**Levels of a selection of transcripts in phloem exudate and their expression in adjacent pod wall tissue**. 1 μg of total RNA isolated from pod tissue and phloem exudates was reverse transcribed followed by real-time PCR analysis. Data are the mean ± standard error of three biological replicates with two technical replicates each. Abb: chlorophyll a/b binding protein (Chl); flowering locus T (FT); small subunit of Rubisco (Rbc); sucrose synthase (SuSy); (SAM) S-adenosyl methionine synthase.

Because the phloem exudates were collected from shallow incisions made to the vasculature it is likely that cells other than SE were also damaged and their contents, including proteins and transcripts, added to those from the SE in the accumulating exudate. To assess the extent that exudate collected from the fruit suture vasculature might be contaminated a number of transcripts were assayed by real time RT-qPCR in both exudate and extracts from the surrounding, non-suture, pod tissue. These included actin, ubiquitin, SAM synthetase, aquaporin, chlorophyll a/b binding protein, small subunit (SSU) of Rubisco, flowering locus T and sucrose synthase. The expression for each transcript in pod tissue extracts was set to 1.0 and the levels of transcript in exudate expressed as a proportion of 1.0 (Figure 3) so that the relative abundance of expression in the pod wall could be compared to that in phloem exudate for each transcript. The pattern of abundance of transcripts relative to one another in exudate did not reflect the relative levels of expression of this group of transcripts in pod tissue.

### Proteins and transcripts identified in lupin phloem exudates are involved in diverse biochemical processes

Proteins and transcripts were grouped by putative function (Figure 2). The largest group of tentatively identified proteins comprised those involved in metabolism (13% general metabolism and 10.7% energy metabolism), followed by protein modification/turnover (9.2%), redox regulation (8.5%), cell structural components (6%) and stress and defence response (6%), with fewer numbers in the other groups (photosynthesis, signalling, transcriptional control and nucleic acid binding). Transcripts coding for proteins with unknown functions formed the largest category (280 sequences, 39% of all ESTs). All proteins with insufficient functional information were classified in this category. The largest groups of transcripts coding for proteins with known functions were metabolism with 15% of all ESTs (11% general metabolism and 4% energy metabolism), protein modification/ turnover with 11% of all ESTs, and redox regulation, signalling and stress response and defence-related with 5% of all ESTs each. A group of 23 sequences (3% of all ESTs) were classified as viral proteins exclusively encoding the polyprotein of bean yellow mosaic virus. Transcripts encoding proteins with multiple or unclear function were grouped as 'unclassified' (4% of all ESTs) (Figure 2). For 31 of the transcripts that were identified their corresponding protein was also detected in phloem exudate (Table 1). Additional file 3 shows the full list of sequenced cDNA clones and their functional classification.

**Table 1:** Proteins for which both the protein and its mRNA were identified in *L. albus *phloem exudate

Protein	Functional categorisation
Thioredoxin	Redox regulation
Cytosolic ascorbate peroxidase	Redox regulation
Glutathione S-transferase	Redox regulation
Monodehydroascorbate reductase	Redox regulation
Dehydroascorbate reductase	Redox regulation
Isoflavone reductase	Stress and defence response
Pathogenesis-related 10	Stress and defence response
Chitinase	Stress and defence response
Ubiquitin extension protein	Protein modification/turnoever
Elongation factor	Protein modification/turnoever
Ubiquitin-conjugating enzyme	Protein modification/turnoever
Ubiquitin-protein ligase	Protein modification/turnoever
Cyclophilin	Protein modification/turnoever
Peptidylprolyl isomerase	Protein modification/turnoever
Proteasome subunit	Protein modification/turnoever
Small subunit of Rubisco	Photosynthesis
Flowering locus T	Signalling
Actin	Cell structural components
Profilin	Cell structural components
Tubulin	Cell structural components
Actin-depolymerizing factor (ADF)	Cell structural components
Malate dehydrogenase	Energy metabolism
Enolase	Energy metabolism
Glyceraldehyde-3-phosphate dehydrogenase	Energy metabolism
Triosephosphate isomerase	Energy metabolism
Fructose-bisphosphate aldolase	Energy metabolism
S-adenosylmethionine synthase	General metabolism
UDP-glucose pyrophosphorylase	General metabolism
UDP-D-glucuronate carboxy-lyase	General metabolism
Aldo/keto reductase	General metabolism
Acireductone dioxygenase	Unclassified

### Cloning small RNAs

Small RNA was isolated from phloem exudate and those in the 18 to 26 nt size class were purified and used to construct a small RNA library. The sequences of 383 small RNAs from the phloem were obtained. These small RNAs ranged from 8 to 35 nt, although the majority were 19 to 23 nt. Comparison with those in the miRBase [28] identified 17 sequences from the phloem library with strong similarity to known miRNAs from seven different families (Table 2). However, many of these were shorter than the similar miRNA in other plants suggesting some may have been degraded during the isolation and cloning procedure.

**Table 2:** Small RNA sequences cloned from *L. albus *phloem exudate and matches of these to miRBase [28]

Name	Sequence	Length (nt)	miRNA	Mis matches^a^
Phl71a	UUUGGAUUGAAGGGAGCUC	19	Oryza sativa and Arabidopsis miR159	0 (2 nt short)
Phl344d	UGGAGAAGCAGGGCACGUG	19	Arabidopsis miR164a,b,c	0 (2 nt short)
Phl51a	UCGGACCAGGCUUCAUUCC	19	Oryza sativa and Arabidopsis miR166	0 (2 nt short)
Phl187c	UCGGACCAGGCUUCAUUCCC	20	Maize miR166c,d,e,f,g,h,i	0
Phl340d	UCGGACCAGGCUUCAUUCC	19	Maize miR166b,c,e,f,g,h,i	0 (1 nt short)
Phl32c	UCGCUUGGUGCAGGUCGGG	19	Arabidopsis miR168a/b	0 (2 nt short)
Phl273a	UCGCUUGGUGCAGGUCGGGUU	21	Arabidopsis miR168a/b	2
Phl79d	UCGCUUGGUGCAGGUCGGGAA	21	Arabidopsis miR168a/b	0
Phl80a	UCGCUUGGUGCAGGUCGGGA	20	Arabidopsis miR168a/b	0 (1 nt short)
Phl324a	UCGCUUGGUGCAGGUCGGGAA	21	Arabidopsis miR168a/b	0
Phl260a	UCGCUUGGUGCAGGUCGGGAA	21	Arabidopsis miR168a/b	0
Phl259c	UCGCUUGGCGCAGGUCGGGA	20	Arabidopsis miR168a/b	1 (1 nt short)
Phl333c	UCGCUUGGCGCAGGUCGGGA	20	Arabidopsis miR168a/b	1 (1 nt short)
Phl339b	UGAGCCGAGGAUGACUUGCCGG	22	Arabidopsis miR169d,e,f,g	1 (1 extra nt)
Phl86d	CUGAAGUGUUUGGGGG	16	Arabidopsis miR395	0 (5 nt short)
Phl86b	UGCCAAGGGAGAGUUGCC	18	Arabidopsis miR399b,c	1 (3 nt short)
Phl224b	CGCCAAAGGGGAGUUGCCC	19	Poplar trichocarpa miR399l Vitis vinifera miR399i	1 (2 nt short)

^a ^(difference in length cf matched miRNA)

### Distribution of miRNAs in white lupin tissues and phloem exudate

**Figure 4:**
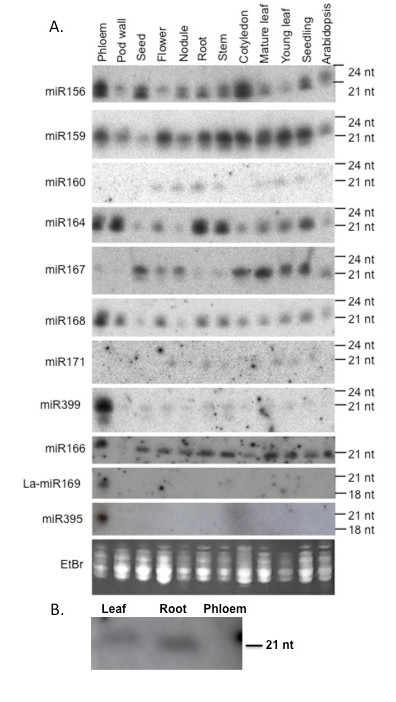
**miRNA present in phloem exudate and lupin tissues**. A) Northern blot analysis of miRNA in various lupin tissues and phloem. Small RNA was extracted from phloem exudate, pod walls, seeds, flowers, nodules, roots, stems, cotyledons, mature leaves, young leaves and three-week-old lupin seedlings and four-week-old *Arabidopsis *seedlings. Small RNA (5 μg) from each sample was separated on a denaturing polyacrylamide gel. After separation, RNA was transferred to Hybond N+ nylon membrane and the membrane was probed with end labelled oligonucleotide probes complementary to microRNAs with conserved sequences in *Arabidopsis *and rice. The position of RNA oligonucleotide standards are indicated on the right. Ribosomal RNA from each sample was visualised by ethidium bromide staining of the polyacrylamide gels and serve as loading controls. B) Northern blot analysis of miR171 in lupin tissues and phloem exudate. Five μg of small RNA extracted from leaf (L), root (R) and phloem exudates (P) of *L. albus *plants were separated on a 15% denaturing polyacrylamide gel, transferred to Hybond-N+ nylon membrane and hybridized to specific ^32^P end-labelled DNA oligonucleotide probes complementary to miR171.

Northern analysis showed 11 miRNAs previously detected in *Arabidopsis *and rice also present in white lupin. Most of the miRNAs were detected in a range of tissues and eight (including miR156, 159, 164, 166, 168, 169-like, 395 and 399) were detected in phloem exudate (Figure 4A). The probe for miR169-like was based on the sequence similar to miR169 that was cloned from lupin. miR169-like, miR395 and miR399 accumulated predominantly in phloem. miR171, which was not detected in phloem in other studies, was not present in lupin phloem either, although the hybridization for this miRNA in Figure 4A is weak. The lack of miR171 in lupin phloem exudate was confirmed using a second blot (Figure 4B). Hybridization with nine other probes for miRNAs was done but none showed a signal in white lupin phloem exudate (results not shown).

In some cases, miRNAs of two sizes were detected. For example, the probe complementary to miR167 recognised RNA approximately 21 nt long in young leaf, seedlings and phloem exudate while RNA detected in seeds, flowers, nodules, roots and stems was only 20 nt. The probe complementary to miR156 detected both 20 and 21 nt RNAs in all tissues. The miR399 in phloem also showed two hybridising bands one at 21 nt and a less significant one at ca 18 nt (Figure 4A). This corresponded to the size of an 18 nt sequence similar to miR399 cloned from phloem exudate.

### Distribution of miRNAs in phloem exudate collected from different sites on the plant

**Figure 5:**
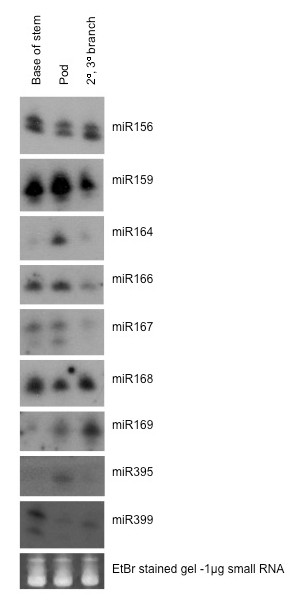
**Distribution of miRNAs in phloem exudate collected from different sites on the plant**. Northern blot assays of 5 μg small RNA extracted from phloem exudate collected from base of the stem, pods and branches of *L. albus *plants. RNA samples were separated on a 15% denaturing polyacrylamide gel, transferred to Hybond-N^+ ^nylon membrane and hybridized to specific ^32^P end-labelled DNA oligonucleotide probes complementary to miR156, miR159, miR164, miR166, miR167, miR168, miR169, miR399 and miR395. Low molecular weight RNA was visualized by ethidium bromide staining to serve as loading control.

Northern analysis of RNA extracted from phloem exudate collected from the base of the stem, developing pods, and from secondary (2°) and tertiary (3°) axillary inflorescence branches (subtended at the top of the plant) was used to determine the distribution of miRNAs within exudate at different sites on the plant. Probes complementary to nine of the miRNAs gave different strength hybridisation signals when bound to RNA from exudate collected from these three sites. Probes complementary to miR164 and miR159 gave the strongest hybridisation signal when bound to pod exudate RNA and weaker signals when bound to RNA from exudate collected at the base and branches at the top of the plant (Figure 5). However, probes complementary to miR168, gave a weaker hybridisation signal in pod exudate RNA than in RNA from exudate at the stem base and from the upper axillary branches (Figure 5). Probes complementary to miR166 and miR167 gave the weakest signals when hybridised to RNA from exudate collected from the top of the plant and stronger signals when hybridised to RNA from pod exudate and exudate from the base of the stem (Figure 5). Consistent with findings from hybridisation experiments to determine the distribution of miRNAs in lupin tissues (Figure 4A), the probe complementary to miR156 gave two strong hybridisation signals in exudate RNA at 20 and 21 nt. The hybridisation signals were approximately equal for all three exudate samples.

**Figure 6:**
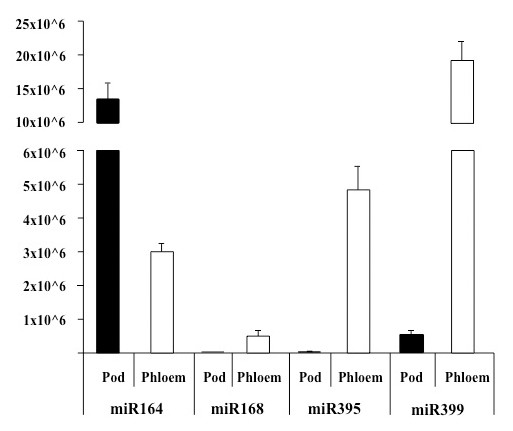
**Absolute quantification of miRNAs in *L. albus *phloem exudate and pod tissue**. 0.5 μg of total RNA isolated from pod tissue and phloem exudate was reverse transcribed using miRNA-specific stem-loop primers followed by real-time PCR analysis performed on a LightCycler480 (Roche Diagnostics) using SYBR^® ^green as the fluorescent dye. Data are the mean ± standard deviation of three biological replicates with two technical replicates each.

The abundance of a number of miRNAs detected in phloem exudate was measured using real time RT-qPCR and the relative abundance compared to adjacent pod tissue was determined (Figure 6). The PCR analysis used primers for miR399d whereas in the northern analysis (Figure 4A) the probe was degenerate and would have picked up a range of miR399s. The relative levels of this group of miRNAs were quite different in the two sources. Except for miR164, which recorded a higher level in the pod compared with phloem (5-fold), miR168, miR395 and miR399 showed much greater abundance in phloem exudate. The enrichments in exudate were 52-, 132- and 39-fold respectively.

**Figure 7:**
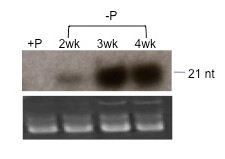
**Accumulation of miR399 in *L. albus *phloem exudate in response to Pi deficiency**. Northern blot of 5 μg small RNA extracted from *L. albus *phloem exudate collected from plants that had been fertilised using a full nutrient solution (+P) or after 2, 3 and 4 weeks after Pi was omitted from the nutrient solution. RNA samples were separated on a 15% denaturing polyacrylamide gel, transferred to Hybond-N^+ ^nylon membrane and hybridised to specific ^32^P end-labelled DNA oligonucleotide probes complementary to miR399. Low molecular weight RNA was visualized by ethidium bromide staining and serves as loading control.

P_i _deprivation in the rooting medium resulted in a significant increase in accumulation of miR399 in phloem exudate collected from the fruits of lupin plants (Figure 7).

## Discussion

### Source of macromolecules in phloem exudate

Unambiguous analysis of the contents of the SE is essential to establish which macromolecules are present in phloem and likely to be translocated. Stylets of sap sucking insects provide the least damaging means for collecting SE contents but to date detailed proteomic and transcriptomic analyses following stylectomy have been restricted to exudate collected from rice and barley [9,15]. An attempt using aphids with castor bean collected a very small amount of exudate and detailed analysis was not possible [5]. However, in a recent study aphid stylet exudate collected from apple stems was analysed and stem-loop RT PCR used to amplify small RNAs, including miRNAs [23]. Thus collecting exudates from dicotyledonous species has relied largely on incisions made to the vasculature [7,5,12,8,11,6] and, while their analysis has shown a broad range of proteins and transcripts, some of which have also been identified in stylet exudate [9], the extent to which they are contaminants from cells surrounding the SE is difficult to determine.

A recent analysis [29] has found that exudate collected from a wound in pumpkin comprises solutes almost exclusively derived from extra fascicular phloem (EFP) and not the main fascicular phloem (FP) system. Thus the metabolite, protein and RNA composition detected in exudate collected from cucurbit species is likely to be derived from SE of the minor EFP and not the major translocation stream (FP). This distinction does not apply to exudates from lupin. There is no structural evidence for spatially distinct phloem systems [30] and quantitative studies of C and N transport, based on assumptions of mass flow of solutes measured in exudates, account for the C and N economy of component organs in the species [31].

Recovery of either proteins or transcripts of Rubisco has been used as a relative measure of contamination of phloem exudate and analysis of exudates collected from wounds to the vasculature shows the presence of both [8,12,6]. The major venation within the sutures of the lupin fruit is bounded by bundle sheath tissue rich in chloroplasts and phloem CC of the suture vasculature showed numerous profiles of intact plastids with limited internal membrane structure [32]. Both these cell types would have been damaged by the incision, their contents contaminating the exudate even though the first drop of exudate that formed was excluded from collection and analysis. Another study [12] used Rubisco as an indicator and concluded that the initial exudate from castor bean contained 12% due to contamination while the subsequent exudate contained only 2%. Large (plastid encoded) and small (nuclear encoded) subunits of Rubisco (gel spots 12, 20 and 36) together with transcripts for the small subunit as well as a number of transcripts for structural components of the photosystems, including chlorophyll a/b binding proteins, were identified in lupin exudate (Table 1 and Additional file 3). Detailed TEM studies of the pod vasculature in white lupin [32] indicate a small number of profiles for P-type plastids in a parietal position in SE, apparently attached to the plasma membrane, and it is possible that these contain some plastid proteins [33]. Interestingly, the observed mass of Rubisco small subunit in spot 12 (Figure 1), ca 6.2 kDa, is lower than the theoretical molecular weight of their closest match which is 15 kDa for Rubisco small subunit of *Phaseolus vulgaris *protein and 20 kDa for Rubisco small subunit from *Glycine max*. Smaller Rubisco proteins were also observed in exudate from *Brassica napus *[8] consistent with the polypeptide being non-functional, having been proteolytically trimmed or catabolised.

Comparative analysis of transcripts in exudate and adjacent pod tissue (Figure 3) indicates that if in fact the transcripts were derived entirely from damaged non SE cells at the wound then the levels of each relative to expression in the pod tissue should be similar. This is clearly not the case indicating that the exudate contained transcripts that were not simply a consequence of contamination. SuSy has been immunolocalised specifically to CC, of both loading and unloading phloem [34] and CC were undoubtedly incised together with the SE in lupin. A SuSy transcript in exudate from castor bean has been identified [12] but in the cereals sampled by stylectomy neither the protein nor its transcript have been documented [15]. While proteomic analysis of lupin exudate did not identify SuSy, this protein has been found in pumpkin exudate [6] and many years earlier SuSy activity in phloem exudate was demonstrated from a cucurbit [35]. The absolute values from the RT-qPCR assays showed that chlorophyll a/b binding protein transcript was the most abundant among the group analysed (results not shown) but the protein was not detected in the proteomic analysis. Like SuSy, the chlorophyll a/b binding protein transcript was also likely to be present in exudate largely, if not solely, as a result of contamination. Using the relative level of the SuSy transcript the contribution of each of the other transcripts analysed from damaged non-SE cells to the exudate was assessed to be 5, 8, 9, 16, 25, 30 and 74% for FT, actin, Rubisco, SAM, ubiquitin, aquaporin and chlorophyll a/b binding protein respectively.

Levels of four of the miRNAs found in phloem exudate compared to adjacent pod tissue (Figure 6) also provide an estimate of the likely levels of contamination. The data indicated that contamination from the damaged non-SE cells at the incision might account for a substantial proportion of miR164. However, this could not have been the case for the other three miRNAs tested, each of which was highly enriched in exudate compared to the surrounding tissue. A relatively low abundance of miR164 compared to miR168, 395 and 399 has also been reported in exudate from *B. napus *[22]. miR171 has been used previously as an indicator of the level of contamination as it has not been detected in other phloem exudates by Northern analysis [21,22] and was not amplified from stylet exudate in apple [23]. A strong hybridisation signal was obtained when the probe was bound to RNA extracted from root tissue and a weaker signal observed when bound to RNA from leaves but there was no detectable signal from phloem exudate (Figure 4B).

Taken together these data indicate that exudate collected from vascular incision in lupin is contributed mainly from SE and that the level of contamination from surrounding damaged tissue is relatively low.

### Proteomic/transcriptomic analyses of exudate

The parallel proteomic and transcriptomic analyses of lupin phloem exudate have identified a vast array of proteins (Additional file 2) and transcripts (Additional file 3) in each case with a lesser group detected in both analyses (Table 1). The fact that the transcript and the product of its translation occur together does not necessarily mean that translation has occurred in the SE. Enucleate SE are not believed to be able to engage in transcription or protein synthesis and macromolecules in SE are formed in phloem CC, traversing the cell interface through plasmodesmata. This belief has recently been challenged by a proteomic analysis of pumpkin phloem exudate [6] in which proteins involved with RNA binding and mRNA translation were identified, and, on this basis, the authors suggested that some proteins in the phloem translocation stream are synthesized in the SE system. Experimental confirmation for this view is lacking and, significantly, another study was unable to show RNA translation in pumpkin phloem exudate using an *in vitro *assay with brome mosaic virus RNA [36]. While proteins involved in protein synthesis were not identified in the exudates from lupin, a small number of transcripts encoding proteins involved in protein synthesis were observed (Additional file 3). The corresponding expressed proteins may have been among the many faintly staining spots that were not sampled from 2D gels and their identity awaits more detailed analysis of the lupin phloem proteome. If protein synthesis in SE can be demonstrated, then long held assumptions about the source of macromolecules in phloem will have to be revised.

#### Protein modification/ turnover

Table 1 shows that both transcripts and proteins associated with ubiquitin-mediated proteolysis were identified in lupin exudate. A proteasome-related protein and components of a ubiquitin-dependent protein degradation pathway have also been identified in *B. napus *exudates by Giavalisco et al. [8]. While these authors suggested ubiquitin-dependent protein sorting could be attributed to these proteins, it has also been hypothesized that the enucleate SE could have retained the capacity for proteolysis [6]. One of the more prominent spots in 2D gels of lupin exudate was a cysteine proteinase inhibitor (spot 1, Figure 1). Proteinase inhibitors have also been identified in phloem exudate from a number of dicotyledon species [5-8,37-39] and it has been suggested they influence the stability of proteins in phloem [8]. Partial degradation of the SE cytoplasm during differentiation must be under tight regulatory control so that the process is inhibited at the appropriate developmental stage and perhaps it is phloem-specific proteinase inhibitors that exert temporal control of this selective autophagy [40].

While the presence of protein degrading enzymes might be expected in SE either in relation to protein turnover [4] or in protecting the phloem against pathogen or insect attack, the presence of their transcripts would not be required unless they had some other role in SE. Perhaps some of these are part of the long distance signalling pathway with a range of as yet unknown functions. A large number of transcripts related to proteinase-inhibitor activity and the ubiquitin-ligase complex has also been found in phloem exudate collected from melon [11].

Proteins and transcripts with chaperone activity, such as cyclophilins (spots 100-103 and 106, Figure 1), were identified together with a peptidyl-prolyl isomerase-like transcript in lupin exudate (Table 1). Cyclophilins, known to occur in SE [41], have also been suggested to play a role in signal processing during development [42] and in protein phosphorylation [38]. It has been proposed that chaperone activity is involved in unfolding, cell-to-cell protein trafficking and refolding of polypeptides on the SE side after import from CC via plasmodesmata [38,43,44]. Cyclophilins are also required for miRNA regulation of gene expression and specifically in the action of miR156 in *Arabidopsis *[45]. miR156 was a prominent species in lupin phloem exudate (Figures 4 and 5) and it is tempting to speculate that it might be translocated together with cyclophilin to some site of action. Ubiquitin extension protein (spot 31, Figure 1), ubiquitin conjugating enzyme-like protein (spot 95, Figure 1) and ubiquitin protein ligase (spot 97, Figure 1) have also been identified in lupin exudate. Each of these could be involved in the transport of proteins synthesized in the CC to the SE, playing a role in maintenance of the enucleate SE.

Spots 74 and 81 (Figure 1) were identified as a UDP-glucose:protein transglucosylase. Such activity could lead to post-translational modification in phloem and might account for the multiple spots on gels that were found for some of the identified proteins. It would be interesting to assess the degree to which glycosylated proteins occurred in these exudates. Several pumpkin phloem exudate proteins appear to be post-translationally modified by phosphorylation and/or glycosylation and their modification seems to be required for the interaction of non-cell-autonomous phloem proteins with chaperones, enabling their movement through plasmodesmata [46]. Protein kinases were present in rice exudate collected by stylectomy together with evidence for Ca^2+^-dependant phosphorylation of proteins [47] as well as in stylet exudate from barley [15]. While both protein kinases and calmodulin were among the transcripts in lupin exudate (Additional file 3) their proteins were not recorded from the 2D-gel analysis. S-adenosylmethionine (SAM) synthase mRNA (Table 1) and protein (spots 70-72, 40-42 and 44; Figure 1), along with methionine synthase (spot 51; Figure 1) were identified in this study. While there may be other fates for methionine and SAM one possibility is in the provision of methyl groups for methylation reactions in SE, possibly associated with post-translational modification of proteins.

#### Cell structural components

Actin (spot 46, Figure 1) and profilin (spot 32, Figure 1) proteins together with their mRNA (Table 1) have been identified in phloem exudate of lupin. Both proteins have been recorded in phloem exudates from a number of monocotyledon and dicotyledon species [8,38] and it has been demonstrated that both are constantly delivered into the SE and are mobile in the translocation stream of castor bean [48]. In the latter profilin was present in a 15-fold molar excess compared to actin [48] and this also appears to be the case in lupin (Figure 1). Profilin is a potent regulator of actin polymerization and its elevated level within the translocation stream may prevent formation of microfilaments [48] by inhibiting actin polymerization [5]. Actin and actin-depolymerizing factors (spots 29, 96 and 121, Figure 1) appear to be part of the common proteins present in pumpkin, rice and Brassica phloem exudates [6] suggesting conservation of actin-dependant processes in phloem including those of legumes. Whether or not there is a dynamic network of cytoskeletal elements that remodel cytoplasmic architecture in response to external and internal stimuli [49] in the mature SE remains to be determined.

#### Redox regulation

The presence of a complete and functional antioxidant defence mechanism in the phloem has been suggested in a number of previous studies [7,50,51]. Proteins involved in cell redox homeostasis, such as glutathione peroxidase (spot 3, Figure 1), ascorbate peroxidase (spot 48, Figure 1), monodehydroascorbate reductase (spot 73, Figure 1) and dehydroascorbate reductase (spots 117 and 129, Figure 1), were all identified in lupin exudate, consistent with the existence of an effective ascorbate-glutathione cycle in phloem. Interestingly, the corresponding transcripts of these proteins were also present (Table 1). Synthesis of nitric oxide has been demonstrated in CC of *Vicia faba *vascular bundles *in vivo *in response to salicylic acid and H_2_O_2 _[52], each of these compounds has been linked in a response pathway to pathogen attack or other stress responses. Thus a very complex relationship between such a pathway and antioxidant defense mechanisms involving glutathione (GSH), for example, must exist in the vasculature as a general reaction to sudden stress and pathogen attack. The GSH levels in cucurbit exudates were as high as 1 mM [53] together with significant rates of GSH reductase activity. Proteins involved in cell redox homeostasis may also play a role in phloem protein stability under prolonged stress and in maintenance of SE integrity by removing ROS over the long life of the SE/CC complex [54].

Thioredoxin (Trx) *h *was identified (spots 33, 55, 108, 109, Figure 1) together with a partial transcript in lupin exudate (Table 1). Importantly, *Trx *mRNA has been identified in phloem exudate collected from rice by stylectomy [13] and a role in phloem protein stability under stress and in maintenance of SE has been attributed to this group of proteins [51,54]. A 13 kDa cytosolic *h*-type Trx moved from CC to adjacent SE in rice [55] and in *Arabidopsis *Trx *h9 *is associated with the plasma membrane and shows intercellular mobility [56]. Interestingly this last study also found that this particular Trx was reduced by GSH rather than NADP-Trx reductase and a loss of function mutant for the gene was severely reduced in growth and development [56]. It was postulated that the cell-to-cell communication by Trx in this case relayed redox information [56] and it is conceivable that a similar role might be important in maintenance of the SE. The implications of the presence of *Trx *transcript in exudate remain to be elucidated. However, *Arabidopsis *mutants with lesions in an *m*-type Trx showed increased callose accumulation and reduced plasmodesmatal permeability [57]. Whether or not Trxs generally are involved in redox regulation of callose deposition and other phloem biochemistry remains to be assessed. However, it has been shown, through integrated biochemical and genetic assays, that the NADPH-dependent Trx system was an effective backup for GSH reductase in *Arabidopsis *[58].

Glutathione S-transferase (GST) (spots 63 and 122, Figure 1) was also present in lupin exudate. This enzyme catalyses a reaction between the thiol group of cysteine in the tripeptide to form a conjugate with xenobiotic molecules (toxins, herbicides etc) rendering them inactive/non-toxic. Perhaps GST functions to conjugate and translocate xenobiotics or endogenous toxins that enter the SE/CC complex. It should be noted that while glutathione is potentially important for these various 'protection' functions it is also a significant form of translocated S in plants [59] and is believed to be the long distance signal in phloem that regulates sulphate ion uptake in roots [60].

#### Stress and defence response

In addition to the group of proteins associated with redox regulation a related category of proteins and transcripts also involved in responses to stress and in defence against pathogens and insect attack were identified in lupin exudate. These included pathogenesis related proteins (PRP), metallothionens and proteins involved in ethylene synthesis.

Identification of a PRP (spots 21 and 22, Figure 1) in the phloem of lupin suggests an active, localised, defence reaction mechanism. It is not clear whether the presence of this protein is constitutive or whether it indicates that the plants from which phloem exudate was collected were responding to a pathogen attack. Although phloem exudate was collected from field-grown plants that appeared healthy, the possibility that pathogens had infected the plants without producing visible symptoms cannot be excluded. In this regard, it may be significant that 10 transcripts, comprising 3% of characterised ESTs, were identified in lupin exudate as those for bean yellow mosaic virus polyprotein. This pathogen is a common disease of lupins in Western Australia and is transmitted by aphids [61]. Phloem from celery petioles has been dissected and mRNA compared from plants with and without feeding aphids [62]. Transcripts for 126 genes increased in plants with aphids and these included transcripts involved in stress responses (metallothionens, catalase, GSH peroxidase, and SAM synthetase among many others). Given that the lupin plants used in this study were exposed to virus infection and probably also aphids it is not surprising that many of the aphid-enhanced transcripts identified previously [62] were also found in the exudate from lupin.

Spots 14 and 15 (Figure 1) were quite prominent and were identified as isoflavone reductase and pterocarpan (or pterocarpin) reductase respectively. These two proteins are NADP-linked oxidoreductases involved in the synthesis of isoflavonoid phytoalexins in legumes [63]. Phytoalexins are also likely to be part of the plant's defences against microbial and herbivore attack but whether or not these defence molecules are present in and translocated by phloem is not known. Isoflavone reductase mRNA was also identified in lupin exudate (Additional file 3).

#### Metabolism

Proteins and transcripts in this category comprised a diverse range involved in carbohydrate (particularly sugar) metabolism, the synthesis and breakdown of organic acids, amino acids and other N-containing substrates, fatty acid and secondary metabolite synthesis and in ATP/adenylate metabolism as well as a host of transferase enzymes. Phloem exudate collected from castor bean contained a full complement of glycolytic intermediates [64] and in a number of proteomic analyses of exudates glycolysis pathway proteins have been identified. These data have led to the generalisation that cytosol-localised glycolysis may be involved with carbohydrate metabolism in SE/CC complexes to produce ATP for phloem loading and glucose for callose formation [65]. However, some glycolytic enzymes may be multifunctional proteins involved in processes other than carbohydrate metabolism [66]. In lupin exudate, proteins involved in glycolysis included fructose-bisphosphate aldolase (spot 75; Figure 1), enolase (spots 35 and 39; Figure 1), glyceraldehyde-3P dehydrogenase (spots 37, 38, 76, 77 and 115; Figure 1), triosephosphate isomerase (spots 47, 66 and 68; Figure 1) and fructokinase (spot 59; Figure 1). Nevertheless, a complete glycolytic pathway was not found and this was also the case for rice phloem collected by stylectomy [9]. Interestingly, the mRNA of many of these proteins was also identified in lupin exudate (Table 1 and Additional file 3).

The SEs in lupin pod suture vasculature show a number of mitochondria with limited cristal structure [32] but the only protein of the Kreb's cycle identified in exudate was malate dehydrogenase (spots 34, 85; Figure 1) together with its transcript (Table 1). While other components of a respiratory pathway may have been present but not detectable on 2D gels the data suggest that mitochondria in SE may not function in ATP-generation. A number of other proteins involved in carbohydrate metabolism were detected in lupin exudate including mannose 6P reductase (spot 84; Figure 1), UDP-D-glucuronate carboxy-lyase (spot 114; Figure 1) and, as noted above, a UDP-glucose:protein transglucosylase. Other enzymes of glucuronate metabolism were not identified but it could be significant that D-glucuronate is a precursor for ascorbate synthesis, another potential metabolite for pathways of redox regulation.

#### Signalling

The question of long distance signalling in plants together with a functional role for phloem has long exercised the minds of plant physiologists. There seems little doubt that the common plant growth regulators (auxin, cytokinins, gibberellins etc) are phloem mobile but even in these cases the connection between their presence in exudates, translocation and mode of action at a 'sink' is not well established. Identification of proteins in phloem exudates has highlighted a possible role for the vasculature in synthesizing some phytohormones *in situ *[39], including jasmonic acid (JA) and ethylene. There is also recent evidence for localization of expression of one of the isopentenyl pyrophosphate transferase alleles (IPT3) in phloem, consistent with localized CK synthesis [67]. As noted above, S-adenosylmethionine (SAM) synthase mRNA and protein along with methionine synthase were identified in lupin exudate. One fate for SAM is synthesis of ethylene from methionine through the intermediates SAM and aminocyclopropane-carboxylate (ACC) [68]. Activities of ACC synthase and ACC oxidase would also be required for *in situ *production of ethylene in SE and neither was detected in lupin exudate. ACC oxidase is common to almost all plant tissues and perhaps it was among the spots that could not be identified or were not sequenced. A further metabolic fate for SAM is in the biosynthesis of polyamines, but there was no evidence for proteins associated with these reactions in lupin exudate.

One of the prominent spots identified on gels of lupin exudate contained a protein that showed high homology to Flowering Locus T-like 1 protein (FT) from *Chenopodium rubrum *(spot 28; Figure 1). Because lupin exudate was collected from developing fruits on plants where flowers were still being fertilized on secondary and tertiary inflorescences, the presence of FT in phloem is not unexpected. These results are consistent with the reported presence of FT proteins in exudate collected from *Brassica napus *[8], *Cucurbita. maxima *[6] and, significantly, by stylectomy from rice [9]. FT proteins and their orthologs act as non-cell-autonomous signals that regulate flowering in *Arabidopsis *[69], rice [70] and cucurbits [71]. FT transcript was not detected using real-time RT-PCR with exudate collected from flowering pumpkin [71] or rice [70] but was identified in (Table 1 and Additional file 3) and readily amplified from lupin exudate (Figure 2). Whether or not FT transcripts or proteins constitute the elusive 'florigen' their presence in phloem exudates from diverse sources, including legumes, lend further support to the idea that each or both are indeed long distance signals.

It has been suggested that certain signals might be transduced by phosphorylation and dephosphorylation reactions in the SE of rice plants [41]. As noted above several proteins in rice phloem exudate, collected by stylectomy, were phosphorylated *in vivo *[41]. Inhibition of protein phosphorylation by a protein kinase inhibitor *in vitro *was consistent with protein kinases existing in the phloem sap of rice plants in a soluble form [41]. No protein kinase proteins were identified in lupin phloem exudate but a small group of transcripts encoding protein kinases and calmodulin was recorded (Additional file 3).

In addition to acting at their site(s) of synthesis and entry into the phloem system, proteins within the SE may themselves play a role in long-distance signalling [38]. While questions about the likely origin of all proteins and transcripts in exudates remain, it is most critical for those for which signalling roles have been postulated. To a degree one critical piece of evidence, yet to be gathered is that either the protein or its transcript is found in exudate collected from insect stylets as well as from incisions to the vasculature.

#### Nucleic acid binding

RNA binding proteins have been characterised in phloem exudates [21,72-74]. Two of the more prominent spots (26 and 27; Figure 1) on gels of lupin exudate were identified as containing a glycine-rich RNA-binding protein (GRP). Other proteomic analyses of exudates have identified these proteins [5,6,8,9]. It has been suggested that some members of this family may be involved in stress responses, as their mRNA levels change following exposure to cold, wounding, hormone treatments and water stress in both plants and animals [75,76]. However, the physiological function of these proteins is still not clear. Expression of *Ccr1 *and *Ccr2*, two members of a class of *Arabidopsis *GRPs, was influenced by cold treatment and circadian rhythm [77] and it was hypothesized that CCR1 could stabilize mRNA species during conditions of cold and in response to other environmental stresses [77]. Other possible functions of these types of protein include pre-mRNA processing, mRNA translation and stability, mRNA repression and/or protein turnover [78]. Although it is quite tempting to speculate that GRPs could form ribonucleotide complexes and translocate RNA, as has been reported for some other RNA binding proteins, to date there are no reports of RNA translocation properties attributed to this group of proteins. GRPs also appear to have a role in floral transition in *Arabidopsis*, specifically in regulation of flowering time [79]. The loss of function mutant, AtGRP7, exhibits a delay in the transition to flowering, whereas the gain of function through ectopic over-expression of AtGRP7 promotes flowering [79]. Some ribosomal proteins and proteins associated with translation also display RNA-binding capacity. The protein and RNA components of a phloem mobile RNA binding protein (RBP50 based) complex in pumpkin have been identified and characterised [72]. Interestingly, one of the phloem transcripts identified as part of the complex was an ethylene response factor also identified in lupin exudate (Additional file 3).

### MicroRNAs in exudate

Following the initial identification of miRNAs in phloem exudate from cucurbits [21] and white lupin [80], a number of studies have suggested that these small RNA species might be phloem-mobile and may have a long-distance signalling role [22,24,25,81]. Systemic signalling could reasonably be expected to be involved in a range of 'source-sink relationships' whether these relate to the partitioning of assimilates, nutrient allocation or in the coordination of development processes, defence or responses to abiotic stress [reviewed in [16]]. A recent compilation identified 13 miRNAs involved in plant responses to drought/salt stress [82]. Eight of these were identified in lupin phloem exudate (Figure 4A) and, importantly, six have been amplified from apple stylet exudate [23].

A unique feature of legumes is their capacity to fix atmospheric N in root nodules and a number of miRNAs have been linked to early stages of nodulation and the subsequent development of mature functional nodules [83]. These include miRNAs associated with hormone responses and include miR156, miR159, miR168, miR169 and miR399, all of which have been found in lupin phloem exudate collected from the downward moving translocation stream (Figures 4A and 5). Nodules are a significant phloem 'sink' for nutrients and especially sugars and it is not unreasonable to expect that this may also apply to macromolecules in phloem. Whether the miRNAs that accumulate differentially in nodules as they develop are due in part to translocation remains to be determined.

Among the 11 miRNAs analysed by northern blot in lupin tissues some were prominent in phloem exudate while others were either absent or much lower than in the other tissues studied (Figure 4A) suggesting that there is a specific spectrum of miRNAs in lupin phloem as seen in *Brassica napus *[25]. Furthermore, this spectrum differed at different sites on the plant where exudate was collected (Figure 5). The most reasonable explanation for the presence of miRNAs in phloem is that they are transported from the adjacent CC. Thus, the fact that the pattern for five miRNAs in exudate collected at three different sites (Figure 5) was not the same suggests either their differential expression in CC at different sites and/or specificity in phloem loading. Interestingly, another study made parallel analyses of miRNAs in stylet exudate and phloem tissue extracts and found that while seven miRNAs were common four that were amplified from the tissue were not detected in exudate lending further support to the idea that transfer to the SE is specific [23]. If miRNAs serve as translocated signals it is not too surprising that the downward-moving (base of stem Figure 5) and upward-moving phloem streams (pods and axillary branches Figure 5) showed differences in miRNA composition. As noted above, lupins offer the possibility of sampling exudate from phloem translocating from 'source' organs of the shoot, including leaflet midribs and petioles, to 'sinks' such as fruits and apices as well as to those of the root system separately [84]. It would be interesting to further exploit this ability by extending the range of miRNAs assayed to include the many more that have now been identified in exudates [22,25].

The question of contamination due to the mode of phloem sampling in lupin, as discussed above for proteins and gene transcripts, applies equally to miRNAs. The data gathered here for four miRNAs in exudate versus the surrounding pod tissue (Figure 6) clearly indicates that three of those assayed were highly enriched in exudate. Although the extent to which exudate might be contaminated cannot be easily estimated it seems likely that miR168, miR395 and miR399 are normal constituents of phloem in lupin while miR164 is not. miR168 was the most abundant cloned miRNA from *B.napus *phloem exudate [22].

The most compelling case for translocation of miRNAs is that of miR399 which increases sharply in both leaf tissue and phloem exudate in response to Pi starvation [22,24,26]. A similar response of miR399 to Pi withdrawal was demonstrated here for lupin (Figure 7) and significantly the most abundant source was the downward moving phloem stream (Figure 5). Recent data has revealed at least four additional miRNAs and several miRNA*s with strong P status-dependent expression in *Arabidopsis *[25] and some of these also show enhanced levels in *B. napus *phloem exudate with Pi limitation. The levels of miR169m in exudate from *B.napus *are decreased in response to decreased N supply [25] and in lupin this miRNA was prominent in exudate (Figure 4A) especially in that collected from newly formed axillary shoots (Figure 5). It will be interesting to examine the status of miR399, miR169 and other miRNAs associated with nutrient homeostasis in translocation streams collected at different sites of lupin under a range of nutrient limitations.

A transcript encoding argonaute4 (AGO4) protein, thought to be involved in RNA silencing [85], was also identified in this study (Additional file 3). The transcript has not been described for other phloem exudates and it is tempting to speculate that it is translocated in phloem to be translated in other parts of the plant where it acts in RNA silencing.

## Conclusions

The results of this study provide the first set of analyses for macromolecules in phloem exudate from a legume. It is reassuring that many of the proteins and transcripts have also been documented in exudates collected from a number of cucurbits [6,39], castor bean [5,12], rapeseed [8,22], and rice [9,13]. Indeed many of the proteins in the 'phloem exudate proteome' derived by comparison among species [6] are conserved in the phloem proteome of lupin. Similarly miRNAs in phloem exudate appear also to be conserved across species though undoubtedly there are many more small RNAs to discover and document in relation to phloem and translocation. While the inventory of macromolecules is a useful starting point, questions about the origin of each in exudate and which are mobile in phloem remain. A critical deficiency is the need for detailed comparative macromolecule analyses of exudate collected by stylectomy from the range of species, including lupin, that have been to date sampled by incisions to the vasculature. The functional significance of each of the proteins, transcripts and small RNAs in phloem also poses new questions about the biochemical features and maintenance of SE. There appears to be specific metabolic components that might also function to support the SE and a wide range of proteins that appear to be involved in 'defence' (either for the plant generally or the phloem in particular). How these relate specifically to either phloem function or systemic signalling is yet to be determined.

## Methods

### Plant material and collection of phloem exudates

Seeds of *Lupinus albus *(L.) *cv*. Kiev mutant (white lupin) were planted in coarse river sand in pots maintained in a naturally lit glasshouse or in the field in a local sandy soil in late May and watered daily. Both the glasshouse- and field-grown plants were inoculated with Bradyrhizobium strain WU425 at sowing. Phloem was collected when nodulated plants were flowering and setting pods. At this time both the nodules and fruit would represent significant sinks for photosynthates [86]. Phloem exudate was collected by making shallow incisions in the lateral and dorsal sutures of fruits, inflorescence stalks and at the base of the stem. The first drop of exudate was discarded and the subsequent exudate stored immediately after collection at -80°C to minimise degradation of proteins, mRNA and miRNA.

### Protein fraction preparation

A proteinase inhibitor cocktail tablet (Roche Applied Science) was added to each 10 ml phloem exudate aliquot. Protein was concentrated on a Centriprep^® ^Centrifugal Filter Device (Ultracel YM-3, 3000MWCO, Millipore) in a Beckman Swinging-bucket rotor at 4°C at 3,000 g. Protein concentration was measured using the method of Bradford [87]. Following column concentration, 700 μl of concentrated phloem sample was precipitated using 9 vol of cold methanol. The sample was incubated overnight at -80°C and then centrifuged at 12,000 g for 30 min at 4°C. The resultant pellet was air dried at room temperature and resuspended in 600 μl 2-D lysis buffer containing 9 M Urea and 2% (w/v) CHAPS (3-[(3-cholamidopropyl)dimethyl-ammonio]-1-propanesulfonate).

### Two-dimensional gel electrophoresis

2-DE was performed as described in a previous study [88]. Gels were stained using colloidal Coomassie Brilliant Blue G-250.

### Mass spectrometric analysis

Protein spots were excised from stained 2D gels, transferred to low protein binding 1.5 ml tubes (LoBind Tubes, Eppendorf) and destained in a solution containing 40% (v/v) acetonitrile (ACN) and 12 mM ammonium bicarbonate. Destained gel spots were dehydrated by rinsing twice with 100% ACN. Gel spots were then dried by vacuum centrifugation for 15 min and in-gel digested using 12 ng/μl of Trypsin at 37°C overnight. N-terminal derivatisation of the digests was done using 4-sulphophenyl isothiocyanate (SPITC) reagent (Sigma) in 20 mM NH_4_HCO_3 _at 55°C for 30 min. Then, triflouroacetic acid (TFA) was added to a final concentration of 0.3%. After derivatisation, samples were pre-treated with PerfectPure C-18 Tips (Eppendorf) to increase MS spectra quality. Protein samples were eluted using 10 mg/ml of matrix (α-cyano-4-hydroxycinnamic acid, Sigma) solution containing 0.1% (v/v) TFA and 70% (v/v) ACN directly onto a MALDI target plate.

Samples were processed using a quadrupole time-of-flight hybrid mass spectrometer (QSTAR XL mass spectrometer) running with the oMALDI ionisation source. The instrument was calibrated with the fragmentation spectrum of Glu-Fibrinogen B (Sigma, molecular weight 1570.67). After obtaining the MALDI-MS spectra, individual peptide ions with a higher number of counts and mass/charge (m/z) values were selected for further fragmentation obtaining product or daughter ion spectra. Partial amino acid sequences were derived manually from the product spectra. These partial sequences were used for protein database searches using the blastp or tblastn algorithm with parameters set to accommodate short input sequences (Expect threshold: 200000; word size: 2; matrix: PAM30) with the Non-redundant protein sequences (nr) or Non-human, non-mouse ESTs (est_others) (limited to *Lupinus *(taxid:3869)) databases respectively at the National Center for Biotechnology Information (NCBI). Matches were considered significant when identity was greater than 85%. Functions were assigned based on matches to a number of proteins of the same function. Where one peptide in a spot had a match over 85% identity, matches of other peptides from the same spot below 85% were considered. Where the closest match was to an EST from a *Lupinus *species this EST sequence was used in a blastx search to identify the function of the closest homologue (with identity over 75%). This function was compared to the function of the match from the blastp search to confirm that the protein encoded by the EST and match from the Non-redundant protein sequences (nr) database had the same function. A match was considered strong if 2 or more peptides from the same spot matched the same sequence.

### Total and mRNA isolation from phloem exudates

Total RNA was isolated from phloem exudate samples using 5 vol of TRIzol^® ^reagent (Invitrogen) per 1.5 volumes of phloem exudates. Then phloem exudate mRNA was isolated from the total RNA sample using a Dynabeads^® ^mRNA DIRECT™ kit (DYNAL BIOTECH).

### cDNA library construction

A cDNA library was constructed from mRNA using the CloneMiner cDNA Library Construction Kit (Invitrogen). cDNA was cloned into pDONR222™ vector (Invitrogen) by recombination and transformed into E. coli ElectroMAX™ DH10B™ T1 Phage-Resistant Competent Cells (Invitrogen). A total of 1063 clones were randomly selected for sequencing. Sequences were edited to trim segments from vector and primers and sequences of poor quality were eliminated. The inserts of 1063 randomly-selected clones were sequenced using M13 primers (Forward: 5'-GTAAAACGACGGCCAG-3' and Reverse: 5'-CAGGAAACAGCTATGAC-3'). The sequences were subjected to BLASTN and BLASTX searches against the NCBI database. Matches with a threshold E value of 10^-10 ^were considered significant.

### Analysis of small RNA by Northern Blot

Small RNA (< 200 bp) was isolated from lupin and Arabidopsis tissue and phloem exudate using the mirVana miRNA isolation kit (Ambion) as described by the manufacturer. RNA was quantified by spectrophotometry. Some samples were precipitated and resuspended in a smaller volume of elution buffer (Ambion mirVana miRNA isolation kit) to increase the RNA concentration. To precipitate, 20 μg of glycogen, 0.1 vol 5M ammonium acetate and 3 vol 100% ethanol was added to the samples and they were incubated overnight at -20°C. RNA was pelleted by centrifugation and the pellet washed in 75% ethanol.

Small RNA (5 μg) was electrophoretically separated on a 15% denaturing polyacrylamide gel and visualised by ethidium bromide staining. RNA was electroblotted onto nylon membranes using the Trans-Blot Electrophoretic Transfer cell (BioRad) and hybridised with ^32^P end-labelled anti-sense DNA probes. DNA oligonucleotide probes were designed using miRNA sequences previously found in Arabidopsis and rice [28] and end-labelled with [γ^32^P] CTP using T4 polynucleotide kinase supplied with the KinaseMax Kit (Ambion). Unincorporated nucleotides were removed from probes by precipitation as described above except precipitated probes were washed in 80% ethanol and resuspended in 100 μl TE. Spin columns (Ambion) were used to remove unincorporated nucleotides. 18, 21 and 24 nt RNA markers were also end-labelled with γ^32^P in this way.

### Cloning Small RNAs

Small RNAs were cloned from total RNA extracted from lupin seedlings and small RNA extracted phloem exudate. Total RNA (approximately 600 μg) was extracted using Trizol reagent (Invitrogen) according to manufactures instructions and small RNA was extracted from approximately 20 ml of Phloem exudate using the mirVana miRNA extraction kit (Ambion). RNA 18-24 nt was gel purified and cloned as described in online protocols [89].

Cloned small RNAs from phloem were sequenced using ABI Big Dye™ terminator V3 and the sequencing reactions were separated and analysed on an ABI Prism 373048 capillary sequencer. To identify potential lupin miRNAs, cloned sequences were blasted against all small RNAs in the miRNA database [28]. FASTA analysis of the small RNAs was then done to identify potential new miRNAs and potential targets of the small RNAs.

## Authors' contributions

CR-M completed the proteomic and transcriptomic analysis of the phloem exudate, studied effect of Pi deficiency on miR399, did real-time analysis of mRNA and miRNAs in phloem exudate and drafted the manuscript. CAA participated in conceiving, design and co-ordination of the project and drafting of the manuscript. AJM and MEJ took part in cloning and analysis of miRNAs from lupin phloem and reviewed the manuscript. PMCS participated in conceiving, design and co-ordination of the project, took part in cloning and analysis of miRNAs, analysis of peptides from lupin phloem and drafting of the manuscript. All authors read and approved the final manuscript.

## Supplementary Material

Additional file 1 **MS/MS product spectra**. An example of MS/MS product spectra obtained after in-gel Trypsin digest of spots 65 (A, B) & 124 (C, D). The sequences derived from the spectra are shown as an insert. Click here for file

Additional file 2 **Peptide sequences identified from *L. albus *phloem exudate protein spots separated by 2D gel electrophoresis and results from Blast searches**. Phloem proteins were separated by 2D-electrophoresis and analysed by partial sequence determination by MS/MS. Although isoleucine (I) and leucine (L) are not distinguishable by mass spectrometry these are shown as present in the database sequences. Click here for file

Additional file 3 **Results of BLASTX search and functional classification of *L. albus *phloem exudate ESTs**. A cDNA library was constructed from mRNA isolated from phloem exudate collected from *L.albus *plants. Clones were sequenced and their identity established using genomic database information. "Unclassified" transcripts had multiple or unclear function. Click here for file
