# Supplementary material for: Macromolecular composition of phloem exudate from white lupin (Lupinus albus L.)
Source: BMC Plant Biol. 2011 Feb 22;11:36. doi: 10.1186/1471-2229-11-36 (PMC3055823; doi:10.1186/1471-2229-11-36)
Supplement: Additional file 1 — MS/MS product spectra. An example of MS/MS product spectra obtained after in-gel Trypsin digest of spots 65 (A, B) & 124 (C, D). The sequences derived from the spectra are shown as an insert. [file 1471-2229-11-36-S1.PDF]

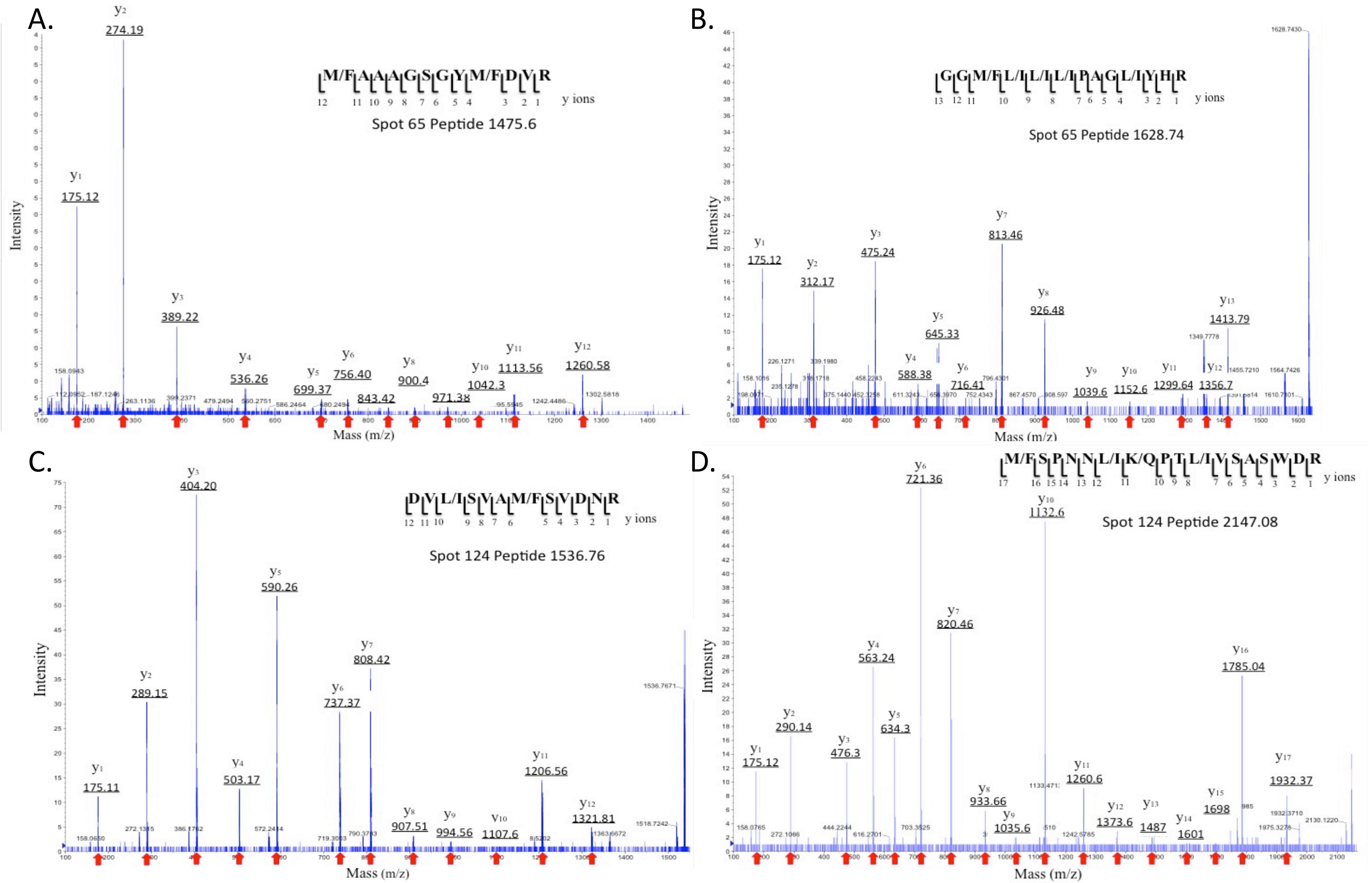

Additional file 1. An example of MS/MS product spectra obtained after in-gel Trypsin digest of spots 65 (A, B) & 124 (C, D). The sequences derived from the spectra are shown as an insert.
