# Supplementary material for: Macromolecular composition of phloem exudate from white lupin (Lupinus albus L.)
Source: BMC Plant Biol. 2011 Feb 22;11:36. doi: 10.1186/1471-2229-11-36 (PMC3055823; doi:10.1186/1471-2229-11-36)
Supplement: Additional file 2 — Peptide sequences identified from L. albus phloem exudate protein spots separated by 2D gel electrophoresis and results from Blast searches. Phloem proteins were separated by 2D-electrophoresis and analysed by partial sequence determination by MS/MS. Although isoleucine (I) and leucine (L) are not distinguishable by mass spectrometry these are shown as present in the database sequences. [file 1471-2229-11-36-S2.PDF]

Additional File 2. Peptide sequences identified from L. albus phloem exudate protein spots separated by 2D gel electrophoresis and results from Blast searches.

Spot No.	Observed protein MW (kDa)	Partial aminoacid sequences that matched in blast search	Protein identification	Acc no.	Organism	e-value	number peptides matched	percent identity
STRESS AND DEFENCE RESPONSE								
1	6	EIAGNQNSLEIDGLAR<br>QNALLEFGR	L.alb_phloem_10-798 *Lupinus albus* phloem cDNA library *Lupinus albus* cDNA with 84% identity to cysteine proteinase inhibitor (Q06445)	GW583953	*Lupinus albus*	1e-07<br>7e-07	2 of 2	100<br>100
		EIAGNQNSLEIDGLAR<br>QNALLEFGR	Cysteine proteinase inhibitor; Cystatin	Q06445	*Vigna unguiculata*	8e-11<br>3e-07	2 of 2	83<br>100
14	9	AIFNKEEDIGTYTIR<br>FFPSEFGNDVDR	s13dLA51F11RT043_527779 *Lupinus albus* L. (white lupin) root *Lupinus albus* cDNA, mRNA sequence with 81% identity to isoflavone reductase-like protein (AF282850)	FG092504	*Lupinus albus*	0.001<br>0.067	2 of 3	100<br>100
		AIFNKEEDIGTYTIR<br>FFPSEFGNDVDR	*Lupinus albus* phloem cDNA library *Lupinus albus* cDNA, mRNA sequence with 85% identity to isoflavone reductase-like protein (XP_002283953)	GW583477	*Lupinus albus*	0.001<br>0.067	2 of 3	100<br>100
		ALFNKEEDIGTYTIR<br>FFPSEFGNDVDR	Unknown similar to NAD(P)H oxidoreductase, isoflavone reductase (pterocarpan reductase)	ACU19092	*Glycine max*	6e-13<br>3e-10	2 of 3	100<br>100
15	19	FFPSEFGNDVDR<br>QVDVVISTVGHLR	*Lupinus albus* phloem cDNA library *Lupinus albus* cDNA, mRNA sequence with 85% identity to isoflavone reductase-like protein (XP_002283953)	GW583477	*Lupinus albus*	1e-08<br>1e-05	2 of 3	100<br>100
		FFPSEFGNDVDR<br>KVDVVISTVGHLL	Pterocarpan reductase (isoflavone reductase)	BAF34843	*Lotus japonicus*	3e-10<br>6e-09	2 of 3	100<br>93
21	8	GIFTFEDESTSTVAPAR	Pathogenesis-related 10	BAB63949	*Lupinus albus*	4e-14	1 of 1	100
22	8	GIFTFEDESTSTVAPAR	Pathogenesis-related 10	BAB63949	*Lupinus albus*	4e-14	1 of 1	100
120	14.5	TFGNGQTLLLNLAGHSR<br>FNDIQTGYSDRR	Class III chitinase	CAA76203	*Lupinus albus*	8e-10<br>4e-08	2 of 2	89<br>83.3
**REDOX REGULATION**								
3	11	FLVDKEGNVVER	s13dLA01C11RT086_521753 *Lupinus albus* L. (white lupin) root cDNA with 90% identity to glutathione peroxidase 1 (AAP69867)	FG089579	*Lupinus albus*	0.15	1 of 2	100
		FLVDKEGNVVER	glutathione peroxidase 1	AAP69867	*Lotus japonicus*	0.015	1 of 2	100
33	6	QIIVVDFTASWCGPCR<br>SVAQDWAVEAMPTF	s13dLA63G11RT042_529251 *Lupinus albus* L. (white lupin) root cDNA with 84% identity to thioredoxin h (CAC36986)	FG093240	*Lupinus albus*	9e-06<br>0.004	2 of 2	100<br>100
		KLIVVDFTASWCGPCR<br>SVAQDWAVEAMPTF	thioredoxin h	CAC36986	*Pisum sativum*	1e-13<br>1e-12	2 of 2	100<br>100
48	15	YAADEDAFFADYTEA<br>HSEGLAHNADNGLDIAVR	s13dLA84A08RT032_531505 *Lupinus albus* L. (white lupin) root cDNA with 94% identity to ascorbate peroxidase (BAA76419)	FG094367	*Lupinus albus*	3e-06<br>1e-06	2 of 3	100<br>79
		YAADEDAFFADYTEA<br>HSEGLAHNADNGLDIAVR<br>LPTDTALLSDPVFKLPVER	cytosolic ascorbate peroxidase 1	ABR18607	*Gossypium hirsutum*	7e-13<br>6e-09<br>3e-08	3 of 3	100<br>73<br>74
55	5	LIVVDFTASWCGPCR	986_F -P proteoid roots 12 and 14 DAE *Lupinus albus* cDNA clone with 87% identity to thioredoxin h (AAZ32865)	CA410717	*Lupinus albus*	9e-05	1 of 3	100
		IIVVDFTASWCGPCR	s13dLA63G11RT042_529251 *Lupinus albus* L. (white lupin) root cDNA with 84% identity to thioredoxin h (CAC36986)	FG093240	*Lupinus albus*	3e-05	1 of 3	100

		LIVVDFTASWCGPCR	thioredoxin h	CAC36986	*Pisum sativum*	2e-06	1 of 3	100
63	14.5	EFAEQLISHLDTFI<br>LYISYLCPFAQR	s13dLA69H05RT017_529991 *Lupinus albus* L.(white lupin) root cDNA with 79% identity to glutathione S-transferase (BAC81649)	FG093610	*Lupinus albus*	0.008<br>0.017	2 of 3	100<br>100
		EFAEQLLSHLDTFL<br>LLGWIEEVNKIDAYTQTR<br>LYISYLCPFAQR	glutathione S-transferase	BAC81649	*Pisum sativum*	8e-12<br>2e-10<br>6e-09	3 of 3	100<br>70.6<br>92
64	14.3	LYTSYICPFAQR	P deficient proteoid roots 12 and 14 days after emergence *Lupinus albus* cDNA clone with 76% identity to soybean In2-1 protein (AF249913)	CA410500	*Lupinus albus*	0.011	1 of 3	100
		LYTSYICPFAQR	In2-1 protein glutathione S-transferase	AAM61679	*Arabidopsis thaliana*	0.011	1 of 3	92
73	29	TVEEYDYLPYFYSR	GW583696 L.alb_phloem_06-421 *Lupinus albus* phloem cDNA library, cDNA with 88% identity to monodehydroascorbate reductase (ABQ41114)	GW583696	*Lupinus albus*	0.003	1 of 2	100
		YVLIGGGVSAGYAAR	BG154008 266 LIN01 *Lupinus luteus* cDNA with 85% identity to unknown similar to monodehydroascorbate reductase (ACU14860)	BG154008	*Lupinus luteus*	0.007	1 of 2	100
		TVEEYDYLPYFYSR<br>YVLIGGGVSAGYAAR	Unnamed protein product similar to monodehydroascorbate reductase I	CBM39039	*Glycine max*	7e-13<br>9e-11	2 of 2	100<br>94
108	5.7	LIVVDFTASWCGPCR<br>TLEADWGVEAMPTF	986_F -P proteoid roots 12 and 14 DAE *Lupinus albus* cDNA clone with 87% identity to thioredoxin h (AAZ32865)	CA410717	*Lupinus albus*	1e-o4<br>0.44	2 of 2	100<br>72
		LIVVDFTASWCGPCR<br>TLEADWGVEAMPTF	Thioredoxin h	CAC36986	*Pisum sativum*	2e-06<br>1.2	2 of 2	100<br>64.3
109	5.7	IIVVDFTASWCGPCR	UWA050 cDNA library from early and middle stages of seed development of narrow leafed lupin *Lupinus angustifolius* cDNA	DT454352	*Lupinus angustifolius*	2e-04	1 of 2	100
		IIVVDFTATWCGPCR	Thioredoxin h	CAC36986	*Pisum sativum*	1e-05	1 of 2	94
117	11.5	ATVGAPNVLGDCPFSQR	Dehydroascorbate reductase	AAL71857	*Nicotiana tabacum*	5e-06	1 of 2	88
122	14	LQPFGKVPAFQDDDLSLFESR	Glutathione transferase	NP_191835	*Arabidopsis thaliana*	5e-07	1 of 4	80.9
129	13	AAADAPNVLGDCPFSQR	L.alb_phloem_08-627 *Lupinus albus* phloem cDNA library, cDNA with 79% identity to DHAR class glutathione transferase DHAR2 (ADB11344)	GW583838	*Lupinus albus*	0.046	1 of 2	100
		AAADAPNVLGDCPFSQR	Dehydroascorbate reductase	AAL71857	*Nicotiana tabacum*	1e-04	1 of 2	89
**PROTEIN MODIFICATION/TURNOVER**								
7	10	TFPQQAGTIR	P deficient normal and proteoid roots 7 and 10 days after emergence Lupinus albus cDNA clone with 96% identity to eukaryotic translation initiation factor 5A2 (ACJ76773)	CA411198	*Lupinus albus*	0.0003	1 of 2	100
31	4	TITLEVESSDTIDNAR<br>IQDKEGIPPDQQR<br>LTIQDKEGIPPDQQR	ubiquitin extension protein	CAA80333	*Lupinus albus*	2e-11<br>2e-11<br>3e-11	3 of 3	87.5<br>100<br>86.6
60	19	VDDHIGVAIAGLTADGR<br>NQYDTDVTTWSPAGR<br>INYCFTYESPLPVGR	Proteasome subunit alpha type, putative	XP_002510414	*Ricinus communis*	8e-12<br>4e-13<br>8e-12	3 of 3	100<br>100<br>94
82	23.5	NLPLVGEVGLGADLVR	Nutrient-reservoir (globulin-like protein)	EEF31270	*Ricinus communis*	0.4	1 of 3	100

86	21	AYLPVIESFGFSSQLR	L.alb_phloem_10-862 *Lupinus albus* phloem cDNA library, cDNA with 94% identity to elongation factor-2 Os02g0519900 (NP_001046972)	GW583988	*Lupinus albus*	1e-07	1 of 1	100
		AYLPVIESFGFSSQLR	Elongation factor-2 Os02g0519900	NP_001046972	*Oryza sativa*	3e-06	1 of 1	100
95	7.5	LLSEPAPGISASPSEENMR<br>NTDKWSPALQIR	s13dLA13F09RT079_522699 *Lupinus albus* L. (white lupin) root cDNA with 98% identity to Ubiquitin-conjugating enzyme E2 (Q9FZ48)	FG090052	*Lupinus albus*	5e-09<br>6e-05	2 of 3	100<br>100
		LLSEPAPGISASPSEENMR<br>NTDKWSPALQIR	Hypothetical protein LOC100500231 similar to ubiquitin-conjugating enzyme	ACU15235	*Glycine max*	5e-16<br>1e-08	2 of 3	100<br>83.3
97	9	GIGDGTVSYGMDDGDDIYMR<br>SWTGTIIGPHNTVHEGR<br>TLGSGGSSVVVPR	Protein binding/ubiquitin-protein ligase	NP_566968<br>NP_001078011	*Arabidopsis thaliana*	3e-18<br>4e-15<br>1e-09	3 of 3	100<br>100<br>100
100	8	IVMELYADTTPR<br>VFFDMAIGGSPVGR	588_F -P proteoid roots 12 and 14 DAE *Lupinus albus* cDNA clone 588 5' with 89% identity to cyclophilin (O49886)	CA410318	*Lupinus albus*	2e-06<br>7e-07	2 of 2	100<br>100
		IVMELYADTTPR<br>VFFDMAIGGSPVGR	Cyclophilin	O49886	*Lupinus luteus*	1e-10<br>8e-09	2 of 2	100<br>79
101	8	VFFDMAIGGSPVGR<br>PRIVSEYADTTPR	588_F -P proteoid roots 12 and 14 DAE *Lupinus albus* cDNA clone 588 5' with 89% identity to cyclophilin (O49886)	CA410318	*Lupinus albus*	7e-07<br>3e-04	2 of 2	100<br>85
		VFFDMAIGGSPVGR<br>PRIVSEYADTTPR	Cyclophilin	O49886	*Lupinus luteus*	8e-09<br>2e-07	2 of 2	79<br>85
102	8	IVMELYADTTPR<br>VFFDMAIGGSPVGR	588_F -P proteoid roots 12 and 14 DAE *Lupinus albus* cDNA clone 588 5' with 89% identity to cyclophilin (O49886)	CA410318	*Lupinus albus*	2e-06<br>7e-07	2 of 2	100<br>100
		IVMELYADTTPR<br>VFFDMAIGGSPVGR	Cyclophilin	O49886	*Lupinus luteus*	1e-10<br>8e-09	2 of 2	100<br>79
103	8	IVMELYADTTPR<br>VFFDMAIGGSPVGR	588_F -P proteoid roots 12 and 14 DAE *Lupinus albus* cDNA clone 588 5' with 89% identity to cyclophilin (O49886)	CA410318	*Lupinus albus*	2e-06<br>7e-07	2 of 2	100<br>100
		IVMELYADTTPR<br>VFFDMAIGGSPVGR	Cyclophilin	O49886	*Lupinus luteus*	1e-10<br>8e-09	2 of 2	100<br>79
106	10	IVIGLYGDDVPQTVENFR	Peptidylprolyl isomerase-like protein	AAM65904	*Arabidopsis thaliana*	1E-08	1 of 3	100
**PHOTOSYNTHESIS**								
12	6.2	RWIPCLEFELEHGFVYR<br>ELDEAKTEYPNSFIR<br>EHNSSPGYYDGR	Ribulose-1,5-bisphosphate carboxylase small subunit rbcS3	AAG24884	*Glycine max*	2e-15<br>1e-09<br>4e-09	3 of 3	100<br>80<br>92
20	8	DTDILAAFR	Ribulose 1,5-biphosphate carboxylase large subunit	CAA93923	*Lupinus angustifolius*	1e-06	1 of 3	100
36	19	TFQGPPHGIQVER	Ribulose 1,5-biphosphate carboxylase large subunit	CAA93923	*Lupinus angustifolius*	8e-11	1 of 2	100
126	14	EYYFLSVLTR<br>SKEVEYVGQVLR	23kDa polypeptide of the oxygen evolving complex of photosystem II	ABQ41909	*Sonneratia alba*	0.39<br>0.94	2 of 2	100<br>92
**NUCLEIC ACID BINDING**								
24	8	GFGFVTFANEQSMR	Putative glycine-rich RNA-binding protein	BAF34340	*Dianthus caryophyllus*	3e-12	1 of 2	100
26	8.5	GFGFVTFANEQSMR<br>NITVNEAQSR	Putative glycine-rich RNA-binding protein	BAF34340	*Dianthus caryophyllus*	3e-12<br>7e-07	2 of 3	100<br>90
27	8	GFGFVTFANEQSMR<br>DAIEGLNGKDLDGR	Putative glycine-rich RNA-binding protein	BAF34340	*Dianthus caryophyllus*	3e-12<br>1e-7	2 of 2	100<br>72
**SIGNALLING**								
28	10.5	TFYTLVMVDADAPSPSNPFLR<br>NFAEINNLIPAAAVYFNCGQR<br>TLFYESPQPSEGIHR	L.alb_phloem_04-213 *Lupinus albus* phloem cDNA library, cDNA with 81% identity to flowering locus T (BAJ33494)	GW583562	*Lupinus albus*	7e-11<br>3e-09<br>7e-07	3 of 3	100<br>96<br>87
		TFYTLVMVDADAPSPSNPFLR<br>NFAEINNLLPAAAVYFNCGQR<br>FTLFYESPQPSEGIHR	Flowering locus T-like 1 protein	ABV56568.1	*Chenopodium rubrum*	8e-16<br>2e-11<br>7e-08	3 of 3	91<br>73<br>80

**CELL STRUCTURAL COMPONENTS**								
29	8.5	KLGSPQGYDDFAASLPADECGR<br>IFFIAWSPDTSR	s13dLA64A04RT016_529275 *Lupinus albus* L. (white lupin) root cDNA with 92% identity to actin depolymerizing factor-like protein (ABC49719)	FG093252	*Lupinus albus*	0.047<br>3e-14	2 of 2	91<br>100
		KLGSPQGYDDFAASLPADECGR<br>IFFIAWSPDTSR	Actin-depolymerizing factor (ADF)	P30174	*Brassica napus*	5e-13<br>6e-10	2 of 2	81<br>92
		YMVIQGEPGVVIR	E631_F -P normal and proteoid roots 7 and 10 DAE *Lupinus albus* cDNA clone E631 5' with 88% identity to profilin (ADB96066)	CA411333	*Lupinus albus*	0.036	1 of 3	92
32	5.7	YMVIQGEPGVVIR	Profilin	ABG88188	*Glycine max*	1e-11	1 of 3	100
46	29	DLYGNIVLSGGSTMFPGIADR<br>SYELPDGQVITIGAER	L.alb_phloem_05-381 *Lupinus albus* phloem cDNA library cDNA with 95% identity to actin (AAD03741)	GW583668	*Lupinus albus*	1e-07<br>2e-04	2 of 4	100<br>100
		DLYGNIVLSGGSTMFPGIADR<br>SYELPDGQVITIGAER<br>IWHHTFYNELR	Actin	AAB40076	*Glycine max*	7e-18<br>3e-13<br>2e-10	3 of 4	100<br>100<br>100
74	25	ELIGPAMYFGLMGDGQPIGR<br>VPEGFDYELYNR	UDP-glucose:protein transglucosylase	O04300	*Pisum sativum*	7e-11<br>0.002	2 of 2	100<br>100
81	25	GPAMYFGLMGDGQPIGR	s13dLA04B07RT061_522099 *Lupinus albus* L. (white lupin) root cDNA, mRNA sequence with 88% identity to UDP-glucose:protein transglucosylase-like (ABA81861)	FG089752	*Lupinus albus*	4e-05	1 of 3	100
		VPEGFDYELYNR	E317_F -P normal and proteoid roots 7 and 10 DAE *Lupinus albus* cDNA clone E317 5' with 92% identity to UDP-glucose:protein transglucosylase-like (ABA81861)	CA411029	*Lupinus albus*	5e-06	1 of 3	100
		GPAMYFGLMGDGQPIGR<br>VPEGFDYELYNR<br>NLSPSFFFNTNYDMER	UDP-glucose:protein transglucosylase	O04300	*Pisum sativum*	7e-15<br>2e-10<br>7e-07	3 of 3	100<br>100<br>79
83	23	LISQIISSLTTSLR	tubulin A	AAX86047	*Glycine max*	1e-10	1 of 2	100
96	8.5	LGENAQGYEDFTASLPADECGR<br>YAVYDFEYLTEGNV<br>IFFIAWSPDTSR	s13dLA66C08RT030_529537 *Lupinus albus* L. (white lupin) root cDNA with 96% identity to actin depolymerizing factor-like protein (ACU13224)	FG093383	*Lupinus albus*	2e-10<br>3e-07<br>3e-06	3 of 3	90<br>100<br>100
		LGENAQGYEDFTASLPADECGR<br>YAVYDFEYLTEGNV<br>IFFIAWSPDTSR	unknown [*Glycine max*] similar to actin depolymerizing factor-like protein (ABC49719)	ACU13224	*Glycine max*	1e-15<br>1e-12<br>8e-11	3 of 3	86<br>100<br>100
121	10	ELDGIQIELQATDPTEMDLDVFR<br>IFFIAWCPDTAR	1099_F -P proteoid roots 12 and 14 DAE *Lupinus albus* cDNA clone 1099 with 95% identity to actin depolymerizing factor-like protein (ACU13224)	CA409561	*Lupinus albus*	2e-11<br>5e-07	2 of 2	100<br>100
		ELDGIQIELQATDPTEMDLDVFR<br>IFFIAWCPDTAR	ADF1 (ACTIN DEPOLYMERIZING FACTOR 1); actin binding	NP_190187	*Arabidopsis thaliana*	5e-13<br>0.12	2 of 2	96<br>83.3
**ENERGY METABOLISM**								
**TCA Pathway**								
34	23	VLVTGAAGQIGYALVPMIAR<br>IVQGLGIDEFSR	Malate dehydrogenase	AAO15574	*Lupinus albus*	2e-16<br>2e-09	2 of 2	100<br>100
85	23.5	PLVTGAAGQIGYALVMNIAR	Malate dehydrogenase	AAO15574	*Lupinus albus*	5E-12	1 of 3	90
**Glycolysis**								
35	35	GNPTVEVDLTLSDGTFAR<br>AAVPSGASTGIYEALELR	894_F -P proteoid roots 12 and 14 DAE *Lupinus albus* cDNA clone 894 5' with 89% identity to enolase (CAB75428)	CA410626	*Lupinus albus*	1e-14<br>8e-09	2 of 2	100<br>100
		GNPTVEVDLTLSDGTFAR<br>AAVPSGASTGIYEALELR	Enolase	CAB75428	*Lupinus luteus*	2e-14<br>6e-14	2 of 2	100<br>100
37	24	GILGYTEDDVVSTDFIGDNR<br>LVSWYDNEWGYSTR<br>VPTVDVSVVDLTVR	Glyceraldehyde-3-phosphate-dehydrogenase	CAI83772	*Lupinus albus*	4e-17<br>4e-13<br>4e-11	3 of 3	100<br>100<br>100

38	24	GILGYTEDDVVSTDFIGDNR<br>LVSWYDNEWGYSTR<br>VPTVDVSVVDLTVR	Glyceraldehyde-3-phosphate-dehydrogenase	CAI83772	*Lupinus albus*	4e-17<br>4e-13<br>4e-11	3 of 3	100<br>100<br>100
39	35	GNPTVEVDLTLSDGTFAR<br>AAVPSGASTGIYEALELR	247_F -P proteoid roots 12 and 14 DAE *Lupinus albus* cDNA clone 247 5', mRNA sequence with 89% identity to enolase (CAB75428)	CA409983	*Lupinus albus*	1e-14<br>8e-09	2 of 2	100<br>100
		GNPTVEVDLTLSDGTFAR<br>AAVPSGASTGIYEALELR	Enolase	CAB75428	*Lupinus luteus*	2e-14<br>6e-14	2 of 2	100<br>100
47	14.5	VIACIGETLEQR<br>WIHENVSADVAASVR<br>KVTPQAQEVHADQLR	L.alb_phloem_05-327 *Lupinus albus* phloem cDNA library cDNA with 90% identity to triosephosphate isomerase (ABA86966)	GW583635	*Lupinus albus*	0.003<br>0.051<br>0.11	3 of 3	100<br>100<br>66
		VIACIGETLEQR<br>WIHENVSADVAASVR<br>KVTPQAQEVHADQLR	Triosephosphate isomerase	ABA86966	*Glycine max*	0.011<br>0.011<br>1.7	3 of 4	100<br>80<br>83
66	14.5	WIHENVSADVAASVR<br>VIACIGETLEQR	L.alb_phloem_05-327 *Lupinus albus* phloem cDNA library, cDNA with 90% identity to triosephosphate isomerase (ABA86966)	GW583635	*Lupinus albus*	0.003<br>0.051	2 of 2	100<br>100
		WIHENVSADVAASVR<br>VIACIGETLEQR	Triosephosphate isomerase	AAT46998	*Glycine max*	2e-10<br>7e-10	2 of 2	80<br>100
68	14.5	VIACIGETLEQR	L.alb_phloem_05-327 *Lupinus albus* phloem cDNA library, cDNA with 90% identity to triosephosphate isomerase (ABA86966)	GW583635	*Lupinus albus*	0.051	1 of 1	100
		VIACIGETLEQR	Triosephosphate isomerase	AAT46998	*Glycine max*	7e-10	1 of 1	100
75	24	EPSELAIHENAYGLAR<br>VAPEVVAEHTVR	L.alb_phloem_09-743 *Lupinus albus* phloem cDNA library cDNA with 91% identity to Fructose-bisphosphate aldolase (O65735)	GW583914	*Lupinus albus*	1e-07<br>1e-04	2 of 2	100<br>92
		EPSELAIHENAYGLAR<br>VAPEVVAEHTVR	Fructose-bisphosphate aldolase	NP_001118453.1	*Arabidopsis thaliana*	3e-06<br>0.12	2 of 2	100<br>92
76	23	LVSWYDNEWGYSTR<br>TEDDVVSTDFIGDNR<br>VPTVDVSVVDLTVR	Glyceraldehyde-3-phosphate-dehydrogenase	CAI83772	*Lupinus albus*	4e-13<br>1e-12<br>4e-11	3 of 3	100<br>100<br>100
77	23	GILGYTEDDVVSTDFIGDNR<br>LVSWYDNEWGYSTR<br>VPTVDVSVVDLTVR	Glyceraldehyde-3-phosphate-dehydrogenase	CAI83772	*Lupinus albus*	4e-17<br>4e-13<br>4e-11	3 of 3	100<br>100<br>100
84	21	SIGISNYDVFLTR	Putative NADPH dependent mannose 6-phosphate reductase	AAM64779	*Arabidopsis thaliana*	0.001	1 of 1	100
114	24	VPTVDVSVVDLTVR	Glyceraldehyde-3-phosphate dehydrogenase	CAI83772	*Lupinus albus*	4e-11	1 of 3	100
115	24	NVSWYDNEWGYSTR<br>VPTVDVSVVDLTVR	Glyceraldehyde-3-phosphate dehydrogenase	CAI83772	*Lupinus albus*	4e-11<br>3e-12	2 of 2	100<br>93.8
**GENERAL METABOLISM**								
**Aminoacid**								
40	30	FVIGGPHGDAGLTGR	S-adenosylmethionine synthetase	ACL14491	*Cicer arietinum*	1e-11	1 of 2	100
41	30	FVIGGPHGDAGLTGR	S-adenosylmethionine synthetase	ACL14491	*Cicer arietinum*	1e-11	1 of 3	100
42	31	FVIGGPHGDAGLTGR[a]<br>TALADGEYYNENGAMVPVR	L.alb_phloem_04-277 *Lupinus albus* phloem cDNA library with s-adenosylmethionine synthetase 4	GW583597	*Lupinus albus*	0.002<br>1e-07	2 of 2	100<br>64.2
		FVIGGPHGDAGLTGR<br>TALADGEYYNENGAMVPVR	S-adenosylmethionine synthetase	AAK71233	*Brassica juncea*	1e-11<br>3e-11	2 of 2	100<br>68.4
44	32	FVIGGPHGDAGLTGR	S-adenosylmethionine synthetase	ACL14491	*Cicer arietinum*	1e-11	1 of 1	100
51	12	YFQTGGEIGFDTYFSMAR	947_F -P proteoid roots 12 and 14 DAE *Lupinus albus* cDNA clone 947 with 88% identity to methionine synthase (AAQ08403)	CA410680	*Lupinus albus*	6e-04	1 of 2	88
		YFQTGGEIGFDTYFSMAR	Methionine synthase	AAQ08403	*Glycine max*	9e-7	1 of 2	83.3

65	13.5	NFFEEHLHTDEEIR<br>GGMIILPAGIYHR<br>LAELGVLSWR<br>FAAAGSGYFDVR	s13dLA70H12RT041_530091 *Lupinus albus* L. (white lupin) root Lupinus albus cDNA, mRNA sequence with 85% identity to acireductone dioxygenase (XP_002517071, ABW34717)	FG093660	*Lupinus albus*	0.009<br>1e-07<br>0.002<br>1e-05	4 of 4	100<br>100<br>100<br>100
		NFFEEHLHTDEEIR<br>GGMIILPAGIYHR<br>LAELGVLSWR<br>FAAAGSGYFDVR	Acireductone dioxygenase	ABW34717	*Solanum tuberosum*	1e-5<br>0.003<br>1.2<br>3.0	4 of 4	100<br>93<br>100<br>84
70	30	FVIGGPHGDAGLTGR<br>TQVTVEYYNSR	s13dLA32D05RT021_526457 *Lupinus albus* L. (white lupin) root cDNA with 99% identity to S-adenosylmethionine synthetase (ACL14491)	FG091843	*Lupinus albus*	0.002<br>0.002	2 of 2	100<br>100
		FVIGGPHGDAGLTGR<br>TQVTVEYYNSR	S-adenosylmethionine synthetase	ACL14491	*Cicer arietinum*	1e-11<br>3e-07	2 of 2	100<br>81.8
71	30	FVIGGPHGDAGLTGR	S-adenosylmethionine synthetase	ACL14491	*Cicer arietinum*	1e-11	1 of 1	100
72	30	FVIGGPHGDAGLTGR	S-adenosylmethionine synthetase	ACL14491	*Cicer arietinum*	1e-11	1 of 1	100
**Sugars and polysaccharides**								
43	33	ATSDLLLVQSDLYTLQDGFVAR<br>VQLLEIAQVPDEHV<br>VLQLETAAGAAIR	L.alb_phloem_02-21 *Lupinus albus* phloem cDNA library, cDNA with 92% identity to UDP-glucose pyrophosphorylase (O64459)	GW583419	*Lupinus albus*	4e-14<br>4e-07<br>4e-06	3 of 3	100<br>100<br>100
		ATSDLLLVQSDLYTLQDGFVAR<br>VQLLEIAQVPDEHV<br>VLQLETAAGAAIR	UDP-glucose pyrophosphorylase	O64459	*Pyrus pyrifolia*	1e-10<br>0.001<br>0.015	3 of 3	96<br>93<br>100
45	33	YLTNSNEIHTFNQSKYPR<br>VLQLETAAGAAIR	L.alb_phloem_02-21 *Lupinus albus* phloem cDNA library, cDNA with 92% identity to UDP-glucose pyrophosphorylase (O64459)	GW583419	*Lupinus albus*	2e-07<br>4e-06	2 of 2	83.3<br>100
		YLTNSNEIHTFNQSKYPR<br>VLQLETAAGAAIR	UDP-Glucose Pyrophosphorylase	2ICX_A	*Arabidopsis thaliana*	6e-06<br>0.001	2 of 2	88<br>100
59	21	VFHYGSISLIVEPCR<br>IVDDQSILEDEPR<br>LPLWPSPEEAR	Fructokinase-like protein	CAD31714	*Cicer arietinum*	5e-06<br>0.015<br>0.037	3 of 3	100<br>93<br>100
114	24	ILVTGGAGFIGSHLVDR	1011_F -P proteoid roots 12 and 14 DAE *Lupinus albus* cDNA clone 1011 5' with 93% identity to UDP-D-glucuronate carboxy-lyase (BAB40967)	CA409475	*Lupinus albus*	7e-10	1 of 3	100
		ILVTGGAGFIGSHLVDR<br>SGFCYVSDLVDGLIR	UDP-D-glucuronate carboxy-lyase	BAB40967	*Pisum sativum*	2e-06<br>6e-04	2 of 3	100<br>87
123	19	SKKLQDLLELAR	s13dLA01E06RT051_521777 *Lupinus albus* L. (white lupin) root cDNA with 81% identity to aldo/keto reductase (XP_002529872)	FG089591	*Lupinus albus*	0.31	1 of 1	100
**Nucleotide**								
56	24	VLPYMDYVFGNETEAR<br>YNVEYIAGGATQNS<br>ANCYAANVIIQR	Adenosine kinase, putative	XP_002531678	*Ricinus communis*	2e-14<br>4e-10<br>1e-08	3 of 3	100<br>92.8<br>92
57	24	ALAYTDFLFGNETEAR<br>VDYMNLSAPFVSEFFR<br>ANCYAANVIIQR	Unknown with similarity to adenosine kinase isoform 1S	ACU19171	*Glycine max*	1e-08<br>3e-11<br>3e-09	3 of 3	92<br>88<br>85
94	7	IIGATNPSQSEPGTIR<br>TFIMIKPDGVQR	886_F -P proteoid roots 12 and 14 DAE *Lupinus albus* cDNA clone 886 5' with 84% identity to nucleoside diphosphate kinase I (AAZ20283)	CA410618	*Lupinus albus*	5e-04<br>0.040	2 of 3	100<br>100
		IIGATNPSQSEPGTIR<br>TFIMIKPDGVQR	Nucleoside diphosphate kinase I	Q39839	*Glycine max*	4e-05<br>0.003		100<br>100
**UNKNOWN**								
2	9	VEVIEYEQVPEYNNR<br>VDEAVYEENVVDVESDR	*Lupinus albus* L. (white lupin) root *Lupinus albus* cDNA, mRNA sequence (not similar to other proteins)	FG089946	*Lupinus albus*	7e-07<br>6e-06	2 of 2	100<br>89

4	8.5	VHEGDWHTAGSVR	P deficient proteoid roots 12 and 14 days after emergence *Lupinus albus* cDNA clone	CA410380.1	*Lupinus albus*	0.0003	1 of 4	84
53	8	VEVIEYEQVPEYNNR	*Lupinus albus* L. (white lupin) root *Lupinus albus* cDNA, mRNA sequence (not similar to other proteins)	FG089946	*Lupinus albus*	7e-07	1 of 3	100
61	23	SPFPFSQTVEAFSYLETGR	BQ441948 LaG312 Differential display fragments *Lupinus albus* cDNA clone G312 similar to auxin-induced protein	BQ441948	*Lupinus albus*	1e-05	1 of 2	100
		SPFPFSQTVEAFSYLETGR<br>VGDEVYGDINR	Auxin-induced protein	AAA87182	*Vigna radiata*	1e-06<br>0.92	2 of 2	90<br>100
62	23	SPFPFSQTVEAFSYLETGR<br>FQVGDEVYGDINR	Auxin-induced protein	AAA87182	*Vigna radiata*	1e-06<br>0.006	2 of 3	90<br>100
80	24	IWPHSFEFR	Unknown protein	ABK23804	*Picea sitchensis*	0.17	1 of 1	100
124	18	FSPNNLQPTIVSASWDR<br>YQSLNAGSIIHSLCFSPNR<br>DVLSVAFSVDNR	Hypothetical protein with similarity to LeArcA2 protein	XP_002281279	*Vitis vinifera*	9e-8<br>0.001<br>0.036	3 of 3	100<br>78<br>100
128	12.5	LQPHPEGGFYTETFR	P deficient proteoid roots 12 and 14 days after emergence *Lupinus albus* cDNA clone	CA409832	*Lupinus albus*	2.1	1 of 1	100
5	6	VDYLEFELEHGFVYR	Unidentified					
6	15	HVDYCNCTGDGVR<br>Q/KNL/IL/IPCVL/IPRR<br>YNVM/FL/IWNR	Unidentified					
8	11	L/IQ/KGHEEGAL/IVL/IGEA<br>L/ITL/IGDAL/IHVGM/FM/FR<br>L/ITL/IGQ/KHCL/IVAL/IHCVTASR<br>LQ/KGVVM/FPSTR<br>EDEQ/KHWL/ICL/IDL/IR	Unidentified					
9	11	L/IQ/KGVVM/FPSTR<br>L/IGDAL/IHVTHL/IR<br>EYGGEAHEGEL/IG<br>L/IQ/KGHEEGVL/IAL/IGEA	Unidentified					
10	10.5	L/IQ/KGHEEGQ/KL/IHRHL/IR<br>L/IPTNVL/ICSTR<br>L/IGDAL/IHVTHL/IR<br>YEGEGHAGL/IM/FGDSR	Unidentified					
11	10.5	AHWTPTGHQ/KL/IQ/KM/F<br>L/IGL/IGEQ/KTNDR	Unidentified					
13	15.5	Q/KRQ/KADYMSQ/KPL/IR<br>NCMVL/IYPL/IR<br>Q/KAWMM/FVYTDER<br>RHCHM/FGCDER<br>NVWTVPPAQ/KNNL/IVR	Unidentified					
16	24	Unfavourable spectra	Unidentified					
17	4.5	EL/IRL/INDWTL/IR	Unidentified					
18	4	Unfavourable spectra	Unidentified					
19	4.5	Unfavourable spectra	Unidentified					
23	8	SGEEQ/KNEDNPR	Unidentified					
25	8	Unfavourable spectra	Unidentified					
30	14	TYVSSGPYM/FESM/FL/INRAR<br>SESSACPSEL/IL/IEDL/IAR<br>Q/KTGDL/IVM/FCTGGR	Unidentified					
49	10	L/IAL/IGGWAL/IHNL/IL/IR<br>NNNDL/ITHAVYM/FQ/KM/FQ/KR<br>NANNEGANHNL/IL/IR<br>ANMHL/ICAL/ISCR	Unidentified					
50	9.5	YDRPDM/FER	Unidentified					

		Q/KL/IPSSHQ/KM/FNCM/FQ/KHR<br>Q/KYDGENPM/FRTGSTTR						
52	10.5	HDQ/KGGYATGGSSM/FL/INRAR<br>MCSGGESVHAL/IGWYGR	Unidentified					
54	7	NSSNSSDYYEQ/KVPEYNNR<br>CAVVSSAEEEL/IERVAR<br>DQ/KCGEEEL/IERVAR<br>CAVCNEEQ/KTAARVAR	Unidentified					
58	23	ACAL/IM/FSHVVQ/KL/IL/IYL/IAR<br>GNYNL/IGEVGL/ISQ/K<br>LVLNPKGFALNCSYMTR	Unidentified					
67	14.5	HHTAVSVNRVVSGM/FL/IRM/FR	Unidentified					
69	16.5	VQ/KSL/IYSQ/KECSSQ/KL/I<br>L/IQ/KNNVPTQ/KNSVGR<br>CNSSQ/KPL/IHL/IRM/FGR	Unidentified					
78	24.5	Unfavourable spectra	Unidentified					
79	25	Unfavourable spectra	Unidentified					
87	18	ESSPQ/KCQ/KCAR<br>NHGNAGCRWL/IRPNTRL/IGR<br>NHGL/IAYSGAHQ/KR	Unidentified					
88	18.5	HAPL/IGL/IGL/IL/ISL/IL/IAHR<br>ESVSMNQ/KHSGAGNPEVAR	Unidentified					
89	42	ACAM/FNVNM/FDQ/KM/FDR<br>ECCGEGVEDGNR<br>WQ/KEYMAEVYR	Unidentified					
90	4.4	M/FNEVVL/INDR<br>DQ/KCCTCVGMDEGGCGR	Unidentified					
91	15.5	Q/KGM/FEDAM/FVYR<br>L/ICEAVVSPEL/IGTR<br>L/IYAAGGAAYDQ/KL/IETSGR<br>Q/KGTVNHGHSGSGR	Unidentified					
92	15.5	WHL/IM/FEGESAR<br>GYGMTGDQ/KL/IE<br>L/IYL/IAL/IVSPEL/IGTR	Unidentified					
93	15.5	EMDGCL/IL/IAHD<br>MMGL/INASNANNVM/FNR<br>Q/KSMHSNNVTQ/KHHMDM/FDR	Unidentified					
98	9.5	M/FQ/KAEM/FPL/IHPR<br>MM/FTCSNYVHNGR	Unidentified					
99	23	HM/FMGDTWGYCR<br>L/IMVVM/FDGTL/IQ/KRR	Unidentified					
104	7.3	VRQ/KPNL/INSDGVT<br>RESDYASNAASTANL/IAGR<br>VL/IL/IVHSGDCVGL/I<br>RDNNYCVVGL/IR	Unidentified					
105	9.5	AHRHVEM/FSSGTM/FVQ/KR<br>L/IDVPQ/KDSM/FVDHVR	Unidentified					
107	11	EDCELYNLGSPVSAVYFNLQR	Unidentified					
110	4.5	Q/KL/IGEM/FTAYTR	Unidentified					
111	16.7	Unfavourable spectra	Unidentified					
112	16.5	SWAVSDDLYEYILETSVYPR	Unidentified					
113	16.7	Unfavourable spectra	Unidentified					
116	12	CCYM/FASDL/IM/FVER<br>HRGL/INCEEL/INYVR	Unidentified					
118	6.5	HGSGSPMYSCATSR	Unidentified					

		SSNGSGEEEEL/IEL/IGRR<br>YQ/KL/IEERNTL/IGRR						
119	6.3	HGMVPYNNGAHSR<br>THNYAEHGHTL/IL/IRASSCNR	Unidentified					
125	15.3	Unfavourable spectra	Unidentified					
127	12.5	YYYNETQAR	unidentified					
130	15.5	HL/ITL/IM/FSL/IL/IGR	Unidentified					

[a] this peptide match is the same for the peptide with the same sequence from spots 40, 41,44,70,71 and 72
