# Supplementary material for: Macromolecular composition of phloem exudate from white lupin (Lupinus albus L.)
Source: BMC Plant Biol. 2011 Feb 22;11:36. doi: 10.1186/1471-2229-11-36 (PMC3055823; doi:10.1186/1471-2229-11-36)
Supplement: Additional file 3 — Results of BLASTX search and functional classification of L. albus phloem exudate ESTs. A cDNA library was constructed from mRNA isolated from phloem exudate collected from L.albus plants. Clones were sequenced and their identity established using genomic database information. "Unclassified" transcripts had multiple or unclear function. [file 1471-2229-11-36-S3.PDF]

Additional File 3. Results of BLASTX search and functional classification of *L. albus* phloem exudate ESTs.

Accession number of closest match	Annotation	Score (bits)	E-value	ESTs per contig
**Energy metabolism**				
ABB46862.2	Enolase, putative, expressed [Oryza sativa (japonica cultivar-group)]	543	1.00E-153	1
BAC77064.1	NADP-specific isocitrate dehydrogenase [Lupinus albus]	528	1.00E-148	1
BAA77604.1	plastidic aldolase NPALDP1 [Nicotiana paniculata]	514	1.00E-144	1
AAL66290.1|AF452450_1	adenosine 5'-phosphosulfate reductase [Glycine max]	500	1.00E-140	1
NP_001078275.1	pyruvate kinase, putative [Arabidopsis thaliana]	473	1.00E-132	1
O65735	Fructose-bisphosphate aldolase, cytoplasmic isozyme [Cicer arietinum]	471	1.00E-131	2
CAA56354.1	NADP dependent malic enzyme [Phaseolus vulgaris]	465	1.00E-129	1
O24301	Sucrose synthase 2 (Sucrose-UDP glucosyltransferase 2) {Pisum sativum]	461	1.00E-128	1
AAO15574.1	malate dehydrogenase [Lupinus albus]	394	1.00E-123	1
ABA86966.1	triosephosphate isomerase [Glycine max]	424	1.00E-117	2
CAI83772.1	glyceraldehyde-3-phosphate-dehydrogenase [Lupinus albus]	413	1.00E-114	2
P80269	NADH dehydrogenase [ubiquinone] iron-sulfur protein 8, mitochondrial precursor (NADH-ubiquinone oxidoreductase 23 kDa subunit) [Solanum tuberosum]	363	5.00E-99	1
AAF01037.1	NADH ubiquinone oxidoreductase PSST subunit [Lupinus luteus]	337	4.00E-91	1
ABA07956.1	glyceraldehyde-3-dehydrogenase C subunit [Glycine max]	311	2.00E-83	1
ABD28700.1	ATP binding , related [Medicago truncatula]	306	1.00E-81	1
BAC81652.1	short-chain alcohol dehydrogenase A [Pisum sativum]	297	4.00E-79	2
Q42961.1	Phosphoglycerate kinase, chloroplast precursor [Nicotiana tabacum]	248	3.00E-64	3
AAY85660.1	cytosolic glucose-6-phosphate isomerase [Helianthus annuus]	220	4.00E-56	1
AAT46998.1	triosephosphate isomerase [Glycine max]	202	1.00E-50	1
CAI53675.1	pyruvate kinase [Glycine max]	147	5.00E-34	1
1F3Y|A	Chain A, Solution Structure Of The Nudix Enzyme Diadenosine Tetraphosphate Hydrolase From Lupinus Angustifolius L.	102	1.00E-20	1
**General metabolism**				
AAL33919.1	UDP-glucose pyrophosphorylase [Amorpha fruticosa]	523	1.00E-147	3
AAA98603.1	mitochondrial aspartate aminotransferase [Glycine max]	525	1.00E-147	1
BAB40967.1	UDP-D-glucuronate carboxy-lyase [Pisum sativum]	514	1.00E-144	1
ABO77438.1	S-adenosyl-L-methionine synthetase [Medicago sativa subsp. falcata]	503	1.00E-141	2
CAJ01707.1	putative S-adenosylhomocystein hydrolase 2 [Hordeum vulgare subsp. vulgare]	504	1.00E-141	1
Q9SP37	Adenosylhomocysteinase (S-adenosyl-L-homocysteine hydrolase) (AdoHcyase) [Lupinus luteus]	495	1.00E-138	2
AAA74441.1	phosphatidylinositol-specific phospholipase C [Glycine max]	478	1.00E-136	1
Q43785	Glutamine synthetase nodule isozyme (Glutamate--ammonia ligase) [Medicago sativa]	477	1.00E-133	1
CAC05439.1	glucose-6-phosphate 1-dehydrogenase [Arabidopsis thaliana]	463	6.00E-129	1
P51850	Pyruvate decarboxylase isozyme 1 (PDC) [Pisum sativum]	461	1.00E-128	1
ABO80948.1	S-adenosylmethionine synthetase [Medicago truncatula]	456	1.00E-127	3

AAO23063.1	ent-kaurenoic acid oxidase [Pisum sativum]	410	1.00E-122	1
1803516A	glycolate oxidase	437	1.00E-121	1
AAW21273.1	glutamine synthetase [Saccharum officinarum]	434	1.00E-120	1
ABO61376.1	serine hydroxymethyltransferase [Populus tremuloides]	434	1.00E-120	1
ABC74567.1	acetoacetyl-CoA thiolase [Picrorhiza kurrooa]	435	1.00E-120	1
CAA53078.1	3-ketoacyl-CoA thiolase B; acetyl-CoA C-acyltransferase [Mangifera indica]	432	1.00E-119	1
NP_188498.1	aspartate/glutamate/uridylate kinase family protein [Arabidopsis thaliana]	430	1.00E-119	1
AAT58365.1	GMPase [Medicago sativa]	427	1.00E-118	1
AAC50014.1	aspartate aminotransferase glyoxysomal isozyme AAT1 precursor [Glycine max]	407	1.00E-115	1
AAK52082.1	nuclease [Nicotiana tabacum]	416	1.00E-114	1
Q9FR44.1	Phosphoethanolamine N-methyltransferase 1 (PEAMT 1) (AtNMT1) [Arabidopsis thaliana]	408	1.00E-112	1
Q96423	Trans-cinnamate 4-monooxygenase (Cinnamic acid 4-hydroxylase) (CA4H) (C4H) (P450C4H) (Cytochrome P450 73) [Glycyrrhiza echinata]	397	1.00E-109	1
ABD33275.2	progesterone 5-beta-reductase, putative [Medicago truncatula]	398	1.00E-109	1
ABN08096.1	Galactose mutarotase-like [Medicago truncatula]	377	1.00E-103	1
CAC10212.1	putative mitochondrial glyoxalase II [Cicer arietinum]	375	1.00E-102	1
AAR13305.1	phytochelatin synthetase-like protein [Phaseolus vulgaris]	372	1.00E-101	1
BAA25187.1	ARG10 [Vigna radiata]	368	1.00E-100	2
AAM60857.1	dihydrolipoamide S-acetyltransferase, putative [Arabidopsis thaliana]	365	2.00E-99	2
ABB29942.1	S-adenosyl methionine synthase-like [Solanum tuberosum]	360	5.00E-98	2
AAX84672.1	aldo/keto reductase AKR [Manihot esculenta]	353	5.00E-96	1
CAO48819.1	unnamed protein product [Vitis vinifera]	350	8.00E-95	1
ABC94943.1	squalene epoxidase [Medicago sativa]	316	8.00E-85	1
BAB33421.1	putative senescence-associated protein [Pisum sativum]	307	4.00E-82	4
AAL74418.2	ATP sulfurylase [Glycine max]	306	1.00E-81	1
CAA74101.1	laccase [Populus trichocarpa]	301	4.00E-80	1
ABD28680.1	Rubber elongation factor [Medicago truncatula]	301	2.00E-80	1
BAC10552.1	nine-cis-epoxycarotenoid dioxygenase4 [Pisum sativum]	288	2.00E-76	1
ABW34717.1	acireductone dioxygenase [Solanum tuberosum]	281	4.00E-74	1
P28002	Caffeic acid 3-O-methyltransferase (S-adenosysl-L-methionine:caffeic acid 3-O-methyltransferase) (COMT) (CAOMT) [Medicago sativa]	268	2.00E-70	1
AAB03852.1	alpha-carboxyltransferase aCT-1 precursor [Glycine max]	257	6.00E-67	1
NP_187342.1	MFP2 (MULTIFUNCTIONAL PROTEIN); enoyl-CoA hydratase [Arabidopsis thaliana]	241	2.00E-62	1
NP_195242.1	O-methyltransferase family 2 protein [Arabidopsis thaliana]	241	4.00E-62	3
BAA88226.1	thiamin biosynthetic enzyme [Glycine max]	239	6.00E-62	2
P43280	S-adenosylmethionine synthetase 1 (Methionine adenosyltransferase 1) (AdoMet synthetase 1) [Solanum lycopersicum]	238	2.00E-61	1
AAK63009.1|AF320025_1	heme oxygenase 3 [Glycine max]	238	1.00E-61	1
Q04593	Phenylalanine ammonia-lyase 2 [Pisum sativum]	238	2.00E-61	1
BAD94178.1	putative myo-inositol 1-phosphate synthase [Arabidopsis thaliana]	219	8.00E-56	2
BAF49299.1	putative glycosyltransferase [Clitoria ternatea]	195	1.00E-48	1

NP_194843.1	glycosyl hydrolase family 17 protein [Arabidopsis thaliana]	192	2.00E-47	1
AAL91002.1	asparagine synthetase [Securigera parviflora]	179	4.00E-44	1
BAB33422.1	putative senescence-associated protein [Pisum sativum]	175	2.00E-42	2
NP_565701.1	lipase class 3 family protein [Arabidopsis thaliana]	169	9.00E-41	1
NP_190412.1	hydrolase, alpha/beta fold family protein [Arabidopsis thaliana]	160	4.00E-38	1
CAA63598.1	glyoxysomal beta-ketoacyl-thiolase [Brassica napus]	156	5.00E-37	1
AAY86360.1	cinnamoyl-CoA reductase [Acacia mangium x Acacia auriculiformis]	149	9.00E-35	1
ABU62755.1	4,5-DOPA dioxygenase extradiol [Nicotiana benthamiana]	147	2.00E-34	1
AAS79665.1	cryptochrome 2A apoprotein [Pisum sativum]	137	5.00E-31	1
AAL89723.1	S-adenosylmethionine decarboxylase [Glycine max]	136	4.00E-31	2
AAC70779.1	granule-bound glycogen (starch) synthase [Astragalus membranaceus]	128	2.00E-28	1
ABW89464.1	glutamine synthetase [Gossypium hirsutum]	119	8.00E-26	1
ABN06032.1	Galactose-binding like [Medicago truncatula]	111	2.00E-23	1
ABO77440.1	S-adenosylmethionine decarboxylase [Medicago sativa subsp. falcata]	72.4	2.00E-11	2
AAB96761.1	ferredoxin-dependent glutamate synthase [Glycine max]	72.8	1.00E-11	1
AAT40304.1	S-adenosylmethionine synthase; SAM synthase [Medicago sativa]	402	1.00E-110	1
**Photosynthesis**				
AAA50172.1	photosystem II type I chlorophyll a/b-binding protein [Glycine max]	514	1.00E-144	6
AAR10886.1	chlorophyll a/b binding protein [Trifolium pratense]	442	1.00E-122	3
AAD27878.1	chlorophyll a/b binding protein CP29 [Vigna radiata]	432	1.00E-119	1
P27521.1	Chlorophyll a-b binding protein 4, chloroplast precursor [Arabidopsis thaliana]	357	7.00E-97	1
ABQ63097.1	photosystem I subunit PsaD [Glycine max]	342	1.00E-92	1
AAA33866.1	ribulose 1,5-bisphosphate carboxylase small subunit [Malus x domestica x Pyrus communis]	300	5.00E-80	1
P08927	RuBisCO large subunit-binding protein subunit beta, chloroplast precursor (60 kDa chaperonin subunit beta) (CPN-60 beta) [Pisum sativum]	298	3.00E-79	1
O65100|FRI3_VIGUN	Ferritin-3, chloroplast precursor	283	8.00E-75	1
BAD97359.1	PsbQ [Nicotiana tabacum]	204	3.00E-51	1
ABI84258.1	photosystem I psaH protein [Arachis hypogaea]	202	2.00E-50	1
P10690	Photosystem II 10 kDa polypeptide, chloroplast precursor [Spinacia oleracea]	156	5.00E-37	1
CAB53034.1	photosystem I subunit XI precursor [Arabidopsis thaliana]	153	4.00E-36	1
CAA96570.1	CP12 [Pisum sativum]	148	3.00E-34	1
P14226|PSBO_PEA	Oxygen-evolving enhancer protein 1, chloroplast precursor (OEE1)	89.7	5.00E-17	1
**Cell structural components**				
Q39445	Tubulin beta chain (Beta-tubulin) [Cicer arietinum]	546	1.00E-154	1
AAF03692.1	actin [Picea rubens]	487	1.00E-136	1
P37392	Tubulin beta-1 chain (Beta-1-tubulin) [Lupinus albus]	469	1.00E-134	1
NP_187818.1	ACT11 (ACTIN-11); structural constituent of cytoskeleton [Arabidopsis thaliana]	451	1.00E-125	1
AAB40079.1	actin [Glycine max]	429	1.00E-119	1
ABN08645.1	Tubulin binding cofactor C [Medicago truncatula]	405	1.00E-111	1
P12459.1	Tubulin beta-1 chain (Beta-1-tubulin) [Glycine max]	394	3.00E-108	1

BAD29243.1	dTDP-D-glucose 4,6-dehydratase-like [Oryza sativa]	390	1.00E-107	1
AAV83799.1	putative actin 1 [Chorispora bungeana]	367	1.00E-100	3
ABX57816.1	alpha tubulin [Picea wilsonii]	306	6.00E-82	1
ABB16985.1	profilin-like protein [Solanum tuberosum]	248	2.00E-64	1
AAU81921.1	profilin [Arachis hypogaea]	221	4.00E-56	1
CAD33929.1	microtubule associated protein [Cicer arietinum]	208	2.00E-52	1
ABA81885.1	profilin-like [Solanum tuberosum]	207	5.00E-52	1
Q941H7	Profilin (Minor allergen Lit c 1) [Litchi chinensis]	158	1.00E-37	1
ABV55999.1	alpha-tubulin 7 [Populus tremuloides]	151	2.00E-35	1
NP_566316.1	kelch repeat-containing protein [Arabidopsis thaliana]	73.6	6.00E-12	1
**Protein modification/turnover**				
ABA12220.1	translation elongation factor 1A-4 [Gossypium hirsutum]	518	1.00E-145	1
ABA12221.1	translation elongation factor 1A-5 [Gossypium hirsutum]	497	1.00E-139	1
ABE91931.1	Proteasome component region PCI [Medicago truncatula]	485	1.00E-135	1
CAC20221.1	ribosomal protein L2 [Glycine max]	466	1.00E-130	2
AAY67798.1	14-3-3 protein [Manihot esculenta]	468	1.00E-130	1
AAB36545.1	ubiquitin-like protein [Phaseolus vulgaris]	444	1.00E-123	2
P35100	ATP-dependent Clp protease ATP-binding subunit clpC homolog chloroplast precursor [Pisum sativum]	435	1.00E-120	2
ABD32840.1	Peptidase C1A, papain; Somatotropin hormone; Peptidase C1, propeptide [Medicago truncatula]	430	1.00E-119	1
ABF18679.1	cysteine protease [Medicago sativa]	415	1.00E-114	1
AAN72085.1	putative aminopeptidase [Arabidopsis thaliana]	391	1.00E-107	1
CAA83548.1	PsHSC71.0 [Pisum sativum]	371	1.00E-103	1
NP_178234.1	60S ribosomal protein L7 (RPL7B) [Arabidopsis thaliana]	370	1.00E-101	1
AAD56020.1	elongation factor-1 alpha 3 [Lilium longiflorum]	369	1.00E-101	2
ABE79560.1	Chaperone DnaK [Medicago truncatula]	358	3.00E-97	1
ABA46772.1	RUB1-conjugating enzyme-like protein [Solanum tuberosum]	353	9.00E-96	2
O65731	40S ribosomal protein S5 [Cicer arietinum]	347	3.00E-94	1
ABE80155.1	Peptidylprolyl isomerase, FKBP-type [Medicago truncatula]	342	2.00E-92	1
CAA04447.1	DnaJ-like protein [Medicago sativa]	335	2.00E-90	2
NP_565477.1	AtRPN1a/RPN1A (26S proteasome regulatory subunit S2 1A); binding [Arabidopsis thaliana]	227	4.00E-90	1
AAR83877	60S ribosomal protein L19 [Capsicum annuum]	330	5.00E-89	2
ABD28502.1	Cyclin-like F-box [Medicago truncatula]	329	1.00E-88	1
CAD29823.2	putative ubiquitin-conjugating enzyme [Populus x canadensis]	325	2.00E-87	1
AAK02067.1	cyclophilin-40 [Arabidopsis thaliana]	322	1.00E-86	1
ABA40437.1	40S ribosomal protein S7-like protein [Solanum tuberosum]	311	3.00E-83	2
O49886	Peptidyl-prolyl cis-trans isomerase (PPIase) (Rotamase) (Cyclophilin) (Cyclosporin A-binding protein) [Lupinus luteus]	312	1.00E-83	5
ABM53472.1	eIF5A [Rosa chinensis]	307	5.00E-82	2
NP_564011.1	UBC36; ubiquitin-protein ligase [Arabidopsis thaliana]	309	2.00E-82	1
AAR83868.1	60S ribosomal protein L12 [Capsicum annuum]	279	2.00E-73	1
P48724	Eukaryotic translation initiation factor 5 (eIF-5) [Phaseolus vulgaris]	275	3.00E-72	1

AAD34458.1	Skp1 [Medicago sativa]	266	8.00E-70	1
CAA80333.1	ubiquitin extension protein [Lupinus albus]	264	4.00E-69	1
ABR25690.1	60S ribosomal protein l7a [Oryza sativa (indica cultivar-group)]	257	3.00E-67	1
ABN08266.1	Poly(ADP-ribose) polymerase, catalytic region [Medicago truncatula]	254	5.00E-66	1
ABF93903.1	60S ribosomal protein L21, putative, expressed [Oryza sativa (japonica cultivar-group)]	245	2.00E-63	1
NP_565053.1	SNF7 family protein [Arabidopsis thaliana]	240	6.00E-62	1
ABG27020.1	SKP1-like b [Medicago truncatula]	234	7.00E-60	1
AAS57912.1	70 kDa heat shock cognate protein 1 [Vigna radiata]	228	3.00E-58	1
O22584	40S ribosomal protein S14 [Lupinus luteus]	223	8.00E-57	1
NP_173692.1	PFL (POINTED FIRST LEAVES); structural constituent of ribosome [Arabidopsis thaliana]	222	2.00E-56	1
Q9M5L0	60S ribosomal protein L35 [Euphorbia esula]	219	7.00E-56	2
NP_195162.2	protease-related [Arabidopsis thaliana]	145	7.00E-54	1
ABJ91230.1	CBL-interacting protein kinase 24 [Populus trichocarpa]	202	6.00E-51	1
ABB55398.1	40S ribosomal protein S10-like [Solanum tuberosum]	206	2.00E-51	1
1909359A	ribosomal protein S19 [Solanum tuberosum]	194	3.00E-48	1
AAP72960.1	putative ribosomal protein L31 [Lactuca sativa]	188	2.00E-46	4
Q06445	Cysteine proteinase inhibitor (Cystatin) [Vigna unguiculata]	171	2.00E-41	1
CAA10612.1	ribosomal protein S14 [Pisum sativum]	156	1.00E-36	1
ABA46751.1	eukaryotic initiation factor 5A4-like protein [Solanum tuberosum]	141	2.00E-32	1
ABY60454.1	putative polyubiquitin [Adonis aestivalis var. palaestina]	133	3.00E-30	1
O04287	Peptidyl-prolyl isomerase FKBP12 (PPIase) (Rotamase) [Vicia faba]	127	2.00E-28	1
O22518	40S ribosomal protein SA (p40) [Glycine max]	111	1.00E-23	1
AAC15418.1	14-3-3 protein homolog [Maackia amurensis]	109	6.00E-23	1
AAL91663.1	60s acidic ribosomal protein [Prunus dulcis]	100	5.00E-20	1
NP_187595.1	CDC48 (CELL DIVISION CYCLE 48); ATPase [Arabidopsis thaliana]	101	1.00E-20	1
AAB88458.1	ribosomal protein S12 [Lupinus albus]	98.6	4.00E-19	1
P25866	Ubiquitin-conjugating enzyme E2-17 kDa (Ubiquitin-protein ligase) (Ubiquitin carrier protein) [Triticum aestivum]	94.4	4.00E-18	1
ABY56104.1	ribosomal protein [Cucumis sativus]	92.8	6.00E-18	1
ABC69274.2	putative DnaJ protein [Camellia sinensis]	89.7	1.00E-16	1
ABD32395.1	Histone-fold/TFIID-TAF/NF-Y [Medicago truncatula]	80.9	4.00E-14	1
**Redox regulation**				
ABQ41114.1	monodehydroascorbate reductase [Vitis vinifera]	507	1.00E-142	1
CAI56334.1	TPA: isoflavone reductase-like protein 5 [Vitis vinifera]	450	1.00E-125	1
AAO13838.1	peroxidase 2 [Lupinus albus]	420	1.00E-116	1
AAG22740.1	allergenic isoflavone reductase-like protein Bet v 6.0102 [Betula pendula]	414	1.00E-114	2
ABM45856.1	cytosolic ascorbate peroxidase [Arachis hypogaea]	382	1.00E-104	1
NP_181836.1	AT-P4H-1 (A. THALIANA P4H ISOFORM 1); oxidoreductase [Arabidopsis thaliana]	370	1.00E-101	1
P32110	Probable glutathione S-transferase (Heat shock protein 26A) (G2-4) [Glycine max]	347	5.00E-94	2
CAM57107.1	Rieske iron-sulphur protein precursor [Glycine max]	345	2.00E-93	1
CAE12168.2	formate dehydrogenase [Quercus robur]	343	8.00E-93	1

ABX79343.1	dehydroascorbate reductase [Vitis vinifera]	340	5.00E-92	1
ABD32912.1	Ferric reductase-like transmembrane component [Medicago truncatula]	341	2.00E-92	1
ABI84254.1	thioredoxin fold [Arachis hypogaea]	291	2.00E-77	2
CAD31838.1	putative quinone oxidoreductase [Cicer arietinum]	290	5.00E-77	1
ABF51006.1	Cu-Zn superoxide dismutase [Arachis hypogaea]	259	1.00E-67	1
AAC83463.1	cationic peroxidase 2 [Glycine max]	236	1.00E-60	2
P49332	Probable glutathione S-transferase parC (Auxin-regulated protein parC) [Nicotiana tabacum]	235	2.00E-60	1
CAM57109.1	Rieske iron-sulphur protein precursor [Zantedeschia aethiopica]	231	2.00E-59	1
BAA10929.1	cytochrome P450 like_TBP [Nicotiana tabacum]	187	6.00E-46	4
T02955	probable cytochrome P450 monooxygenase - maize (fragment) [Zea mays]	144	3.00E-41	1
BAA76419.1	ascorbate peroxidase [Cicer arietinum]	144	2.00E-33	1
CAJ43614.1	monodehydroascorbate reductase [Plantago major]	141	4.00E-32	2
CAA10132.1	superoxide dismutase [Cicer arietinum]	125	2.00E-27	1
BAD18377.1	type 2 metallothionein [Glycine max]	105	2.00E-21	1
ABQ44281.1	metallothionein type 2 [Sesbania drummondii]	93.6	4.00E-18	2
CAJ38394.1	cytochrome b5 reductase [Plantago major]	77.4	3.00E-13	1
**Stress and defence response**				
NP_188317.2	chitinase [Arabidopsis thaliana]	498	1.00E-139	2
CAO41879.1	leucine-rich repeat resistance protein-like protein	369	1.00E-100	1
BAD86819.1	hypersensitive-induced response protein [Lotus japonicus]	329	9.00E-89	1
BAB63949.1	pathogenesis-related 10 [Lupinus albus]	313	7.00E-84	7
AAF15296.2	lipoxygenase [Phaseolus vulgaris]	309	1.00E-82	2
P16148|PLZ12_LUPPO	Protein PPLZ12- Lupinus polyphyllus	236	1.00E-60	1
AAO33591.1	putative early light induced protein [Arachis hypogaea]	220	6.00E-56	1
AAT06600.2	dehydrin [Lupinus albus]	203	8.00E-51	3
AAD50376.1	ripening related protein [Glycine max]	203	1.00E-50	4
CAB44031.1	lectin [Glycine max]	191	4.00E-47	1
CAA03926.1	PR-10 protein [Lupinus albus]	158	1.00E-37	1
AAK73280.1	drought-induced protein [Retama raetam]	144	5.00E-33	5
AAB18970.2	lipoxygenase [Phaseolus vulgaris]	117	3.00E-25	2
AAP23944.1	leucine-rich repeat protein [x Citrofortunella mitis]	108	1.00E-22	1
ABB29467.1	salt-tolerance protein [Glycine max]	96.3	7.00E-19	1
NP_190693.1	senescence/dehydration-associated protein-related [Arabidopsis thaliana]	74.3	2.00E-12	1
AAZ85353.1	putative submergence induced protein 2-like [Solanum ochranthum]	75.1	3.00E-12	1
CAB85628.1	putative ripening-related protein [Vitis vinifera]	75.1	2.00E-12	1
BAE48663.1	Pm52 [Prunus mume]	71.2	4.00E-11	1
AAR26524.1	abscisic stress ripening-like protein [Glycine max]	71.2	8.00E-11	3
**Nucleic acid binding**				
ABN09109.1	Helicase, C-terminal [Medicago truncatula]	492	1.00E-137	1
NP_564150.1	F-box family protein [Arabidopsis thaliana]	475	1.00E-132	1

ABD32851.1	Helicase, C-terminal; Zinc finger, CCHC-type; GUCT [Medicago truncatula]	452	1.00E-125	1
AAG10600.1	MYB-related transcription factor PHAN1 [Pisum sativum]	384	1.00E-105	1
P48513	Transcription initiation factor IIB (General transcription factor TFIIB) [Glycine max]	382	1.00E-104	1
AAX13298.1	MADS box protein SEP3 [Lotus corniculatus var. japonicus]	327	4.00E-88	2
NP_172336.3	ATRX/CHR20; ATP binding / DNA binding / helicase [Arabidopsis thaliana]	286	8.00E-76	1
ABH02875.1	MYB transcription factor MYB123 [Glycine max]	247	8.00E-64	2
O49289	Putative DEAD-box ATP-dependent RNA helicase 29 [Arabidopsis thaliana]	246	1.00E-63	1
NP_001078676.1	RNA and export factor-binding protein, putative [Arabidopsis thaliana]	238	3.00E-61	1
ABS18448.1	WRKY55 [Glycine max]	221	6.00E-56	1
ABN06047.1	Zinc finger, RING-type; Transcription factor jumonji, jmjC; Zinc finger, C2H2-type [Medicago truncatula]	216	1.00E-54	1
NP_186796.1	ATHB-1 (Homeobox-leucine zipper protein HAT5); transcription factor [Arabidopsis thaliana]	211	6.00E-53	1
NP_180427.1	ATP binding / ATP-dependent helicase/ nucleic acid binding [Arabidopsis thaliana]	204	6.00E-51	1
ABI34666.1	bZIP transcription factor bZIP124 [Glycine max]	200	1.00E-49	1
CAL25353.1	ACBF-like dna binding protein [Platanus x acerifolia]	190	7.00E-47	1
AAA82062.1	eukaryotic release factor 3	189	1.00E-46	1
AAG13810.1	PSTVd RNA-binding protein Virp1a [Lycopersicon esculentum]	181	3.00E-44	1
BAE71188.1	BEL1-like homeodomain transcription factor [Trifolium pratense]	179	2.00E-43	1
P26585	HMG1/2-like protein (Protein SB11) [Glycine max]	164	5.00E-39	1
AAA88792.1	nucleosome assembly protein 1 [Glycine maxima]	117	4.00E-38	1
NP_974373.1	MIF2 (MINI ZINC FINGER 2); DNA binding [Arabidopsis thaliana]	130	4.00E-29	1
ABD28504.1	Nucleosome assembly protein (NAP) [Medicago truncatula]	117	8.00E-25	1
AAZ86071.1	MADS-box protein [Glycine max]	111	2.00E-23	1
AAQ96342.1	putative ethylene response factor ERF3b [Vitis aestivalis]	102	3.00E-20	1
NP_190149.1	RNA recognition motif (RRM)-containing protein [Arabidopsis thaliana]	103	1.00E-20	2
NP_177360.1	PATL1 (PATELLIN 1); transporter [Arabidopsis thaliana]	90.9	9.00E-17	1
ABH02837.1	MYB transcription factor MYB81 [Glycine max]	79	2.00E-13	1
**Transport**				
AAL58570.1	vacuolar processing enzyme 2 [Glycine max]	483	1.00E-135	1
NP_200125.1	CNGC1 (CYCLIC NUCLEOTIDE GATED CHANNEL 1) [Arabidopsis thaliana]	454	1.00E-126	1
CAA11025.1	aquaporin [Lupinus albus]	446	1.00E-123	3
AAL66293.1	phosphate transporter [Glycine max]	425	1.00E-117	1
Q9FY14	Probable aquaporin TIP-type (MtAQP1) [Medicago truncatula]	392	2.00E-107	2
NP_569004.1	nucleotide-sugar transporter family protein [Arabidopsis thaliana]	313	1.00E-100	1
ABB02396.1	temperature-induced lipocalin [Medicago truncatula]	339	1.00E-91	1
CAD56216.1	transportin-like protein [Cicer arietinum]	324	4.00E-87	1
CAA98170.1	RAB7C [Lotus japonicus]	313	6.00E-84	1
AAF21428.2|AF165422_1	salt-induced AAA-Type ATPase [Mesembryanthemum crystallinum]	298	4.00E-79	1
BAA25753.1	Ca2+/H+ exchanger [Vigna radiata]	258	2.00E-67	1
NP_177419.1	ATTIM23-2 (Arabidopsis thaliana translocase inner membrane subunit 23-2); protein translocase	158	3.00E-37	1
ABE68718.1	putative aquaporin [Arachis hypogaea]	149	1.00E-34	1

YP_001648758.1	ATP synthase subunit 6 [Mycosphaerella graminicola]	135	3.00E-30	1
ABD32809.1	Vacuolar (H+)-ATPase G subunit; KH, prokaryotic type [Medicago truncatula]	121	3.00E-26	1
ABD33158.1	Syntaxin, N-terminal [Medicago truncatula]	102	1.00E-20	1
NP_182033.1	SEC61 BETA (suppressors of secretion-defective 61 Beta); protein transporter [Arabidopsis thaliana]	85.9	1.00E-15	1
**Signalling**				
BAA92699.1	type 2A protein phosphatase-3 [Vicia faba]	555	1.00E-157	1
ABC61505.1	AGO4-2 [Nicotiana benthamiana]	510	1.00E-143	3
AAU89742.1	serine/threonine protein kinase-like [Solanum tuberosum]	479	1.00E-134	1
Q9SWF9|ZFNL_PEA	Zinc finger CCCH domain-containing protein ZFN-like	475	1.00E-132	1
NP_190753.2	CPK13 (calcium-dependent protein kinase 13) [Arabidopsis thaliana]	461	1.00E-128	1
AAA53276.1	GTP-binding protein [Pisum sativum]	436	1.00E-121	1
NP_192169.1	ATMLO1/MLO1 (MILDEW RESISTANCE LOCUS O 1); calmodulin binding [Arabidopsis thaliana]	431	1.00E-119	1
Q39817	Calnexin homolog precursor [Glycine max]	413	1.00E-114	1
BAA02113.1	GTP-binding protein [Pisum sativum]	414	1.00E-114	1
P51139	Glycogen synthase kinase-3 homolog MsK-3 [Medicago sativa]	404	1.00E-111	1
AAQ72787.1	putative GTP-binding protein [Cucumis sativus]	398	1.00E-109	1
NP_179889.1	casein kinase II alpha chain, putative [Arabidopsis thaliana]	385	1.00E-105	1
NP_191645.1	guanine nucleotide exchange family protein [Arabidopsis thaliana]	366	1.00E-100	1
AAC33305.1	fiber annexin [Gossypium hirsutum]	344	2.00E-93	1
NP_195815.3	ATP binding / kinase/ protein serine/threonine kinase [Arabidopsis thaliana]	319	1.00E-85	1
BAA92697.1	type 2A protein phosphatase-1 [Vicia faba]	313	4.00E-84	1
ABJ74170.1	histidine kinase 1 [Lupinus albus]	306	1.00E-81	1
NP_190767.1	protein kinase family protein [Arabidopsis thaliana]	303	1.00E-81	1
Q5J907	Translationally-controlled tumor protein homolog (TCTP) [Elaeis guineensis]	303	7.00E-81	4
AAM12880.1	GTP-binding protein [Helianthus annuus]	285	1.00E-75	1
AAO49473.1	putative serine/threonine kinase [Vitis vinifera]	274	4.00E-72	1
AAM83095.1	SOS2-like protein kinase [Glycine max]	253	8.00E-66	2
AAM65586.1	receptor protein kinase-like protein [Arabidopsis thaliana]	248	3.00E-64	1
AAG28503.1	hexokinase [Citrus sinensis]	246	2.00E-63	2
BAD01612.1	flowering locus T [Populus nigra]	228	3.00E-58	1
NP_174810.1	ANNAT1 (ANNEXIN ARABIDOPSIS 1); calcium ion binding / calcium-dependent phospholipid binding [Arabidopsis thaliana]	206	1.00E-51	1
ABJ91228.1	CBL-interacting protein kinase 22 [Populus trichocarpa]	197	7.00E-49	1
CAC24474.1	GTP binding protein [Cichorium intybus x Cichorium endivia]	194	4.00E-48	1
AAP72282.2	calcium-dependent calmodulin-independent protein kinase isoform 2 [Cicer arietinum]	186	8.00E-46	1
P54766	GTP-binding nuclear protein Ran1B [Lotus japonicus]	147	2.00E-34	1
ABN08208.1	Remorin, C-terminal region [Medicago truncatula]	97.4	3.00E-19	1
BAA82130.1	acid phosphatase [Lupinus albus]	417	4.00E-115	1
**Viral proteins**				
AAB97459.1	nuclear inclusion B [bean yellow mosaic virus]	562	1.00E-159	4
P17765	Genome polyprotein [Bean yellow mosaic virus]	532	1.00E-150	2

AAF00522.1	polyprotein [bean yellow mosaic virus]	529	1.00E-148	3
AAB37237.1	polyprotein [Bean yellow mosaic virus]	513	1.00E-144	3
ABG33691.1	polyprotein [White lupin mosaic virus]	508	1.00E-142	6
BAE96599.1	polyprotein [Bean yellow mosaic virus]	491	1.00E-137	1
AAF00524.1	polyprotein [bean yellow mosaic virus]	487	1.00E-136	1
ABM69145.1	coat protein [Bean yellow mosaic virus]	371	1.00E-101	1
NP_734173.1	P1 protein [Bean yellow mosaic virus]	212	1.00E-53	1
CAO02584.2	polyprotein [Bean yellow mosaic virus]	106	4.00E-22	1
**Unclassified**				
CAE45585.1	coatomer alpha subunit-like protein [Lotus japonicus]	464	1.00E-129	1
NP_001062159.1	Os08g0500700 [Oryza sativa (japonica cultivar-group)]	355	2.00E-96	1
BAE71192.1	putative Asp1 [Trifolium pratense]	336	1.00E-90	1
NP_001046690.1	Os02g0321900 [Oryza sativa (japonica cultivar-group)]	322	2.00E-86	1
NP_001064799.1	Os10g0465800 [Oryza sativa (japonica cultivar-group)]	312	1.00E-83	2
CAA72315.1	putative 21kD protein precursor [Medicago sativa subsp. x varia]	281	3.00E-74	1
AAM21317.1|AF373100_1	auxin-regulated protein [Populus tremula x Populus tremuloides]	267	6.00E-70	2
NP_189128.2	SEC14 cytosolic factor, putative / phosphoglyceride transfer protein, putative [Arabidopsis thaliana]	253	8.00E-66	1
NP_001046972.1	Os02g0519900 [Oryza sativa (japonica cultivar-group)]	251	3.00E-65	1
CAH66528.1	H0502B11.8 [Oryza sativa (indica cultivar-group)]	232	2.00E-59	1
NP_001065830.1	Os11g0163100 [Oryza sativa (japonica cultivar-group)]	217	6.00E-55	1
NP_172636.1	pentatricopeptide (PPR) repeat-containing protein [Arabidopsis thaliana]	214	5.00E-54	1
ABC70464.1	CASTOR protein [Glycine max]	212	2.00E-53	1
NP_001045723.1	Os02g0122000 [Oryza sativa (japonica cultivar-group)]	201	3.00E-50	1
BAB82450.1	PBng143 [Vigna radiata]	142	6.00E-33	1
AAM62421.1|AF515795_1	Drm3 [Pisum sativum]	126	8.00E-28	2
ABW74471.1	auxin-repressed protein [Paeonia suffruticosa]	81.3	6.00E-26	1
ABR25977.1	osiaa30-auxin-responsive aux/iaa gene family member [Oryza sativa (indica cultivar-group)]	114	2.00E-24	1
AAD32146.1|AF123508_1	Nt-iaa28 deduced protein [Nicotiana tabacum]	114	5.00E-24	1
ABN08340.1	ZIM [Medicago truncatula]	166	2.00E-39	1
AAD09514.1	GMFP5 [Glycine max]	159	3.00E-37	1
AAG33924.1	auxin-repressed protein [Robinia pseudoacacia]	66.6	5.00E-10	1
ABQ81923.1	aminotransferase 2 [Cucumis melo]	241	2.00E-62	1
ABC46708.1	chloroplast photosystem II 10 kDa protein [Arachis hypogaea]	196	7.00E-49	2
NP_198992.2	cell cycle control crn (crooked neck) protein-like [Arabidopsis	168	4.00E-40	1
Q96453|1433D_SOYBN	14-3-3-like protein D (SGF14D)	459	1.00E-127	3
ABA54865.1	putative 3-deoxy-D-arabino-heptulosonate 7-phosphate synthase 3	413	1.00E-114	1
**Unknown**				
CAO44871.1	unnamed protein product [Vitis vinifera]	533	1.00E-150	1
CAO18187.1	unnamed protein product [Vitis vinifera]	528	1.00E-148	1
CAO66720.1	unnamed protein product [Vitis vinifera]	514	1.00E-144	1

CAO65935.1	unnamed protein product [Vitis vinifera]	503	1.00E-141	1
CAA10289.1	hypothetical protein [Cicer arietinum]	491	1.00E-137	2
CAO39286.1	unnamed protein product [Vitis vinifera]	471	1.00E-131	1
CAO44803.1	unnamed protein product [Vitis vinifera]	469	1.00E-130	1
CAO65211.1	unnamed protein product [Vitis vinifera]	458	1.00E-127	1
BAA88228.1	thiamin biosynthetic enzyme [Glycine max]	457	1.00E-127	1
CAO49304.1	unnamed protein product [Vitis vinifera]	451	1.00E-125	1
CAN63254.1	hypothetical protein [Vitis vinifera]	451	1.00E-125	1
CAO70618.1	unnamed protein product [Vitis vinifera]	425	1.00E-123	1
CAO21857.1	unnamed protein product [Vitis vinifera]	438	1.00E-121	1
CAO43786.1	unnamed protein product [Vitis vinifera]	437	1.00E-121	1
CAO40038.1	unnamed protein product [Vitis vinifera]	422	1.00E-116	1
CAO15636.1	unnamed protein product [Vitis vinifera]	409	1.00E-113	1
CAO43989.1	unnamed protein product [Vitis vinifera]	393	1.00E-108	1
CAO60892.1	unnamed protein product [Vitis vinifera]	387	1.00E-106	1
ABK96599.1	unknown [Populus trichocarpa x Populus deltoides]	386	1.00E-105	1
CAO65376.1	unnamed protein product [Vitis vinifera]	382	1.00E-104	1
CAN64994.1	hypothetical protein [Vitis vinifera]	381	1.00E-104	1
CAO15192.1	unnamed protein product [Vitis vinifera]	376	1.00E-103	1
CAO48194.1	unnamed protein product [Vitis vinifera]	373	1.00E-102	1
CAO61388.1	unnamed protein product [Vitis vinifera]	370	1.00E-101	1
ABD32814.1	hypothetical protein MtrDRAFT_AC148995g28v2 [Medicago truncatula]	366	1.00E-100	1
CAO23556.1	unnamed protein product [Vitis vinifera]	369	1.00E-100	1
ABK95228.1	unknown [Populus trichocarpa]	365	1.00E-99	1
CAO15903.1	unnamed protein product [Vitis vinifera]	355	2.00E-96	1
CAO45373.1	unnamed protein product [Vitis vinifera]	353	5.00E-96	1
CAO43035.1	unnamed protein product [Vitis vinifera]	351	3.00E-95	1
CAO71749.1	unnamed protein product [Vitis vinifera]	350	7.00E-95	2
CAO61488.1	unnamed protein product [Vitis vinifera]	298	9.00E-95	1
ABK96295.1	unknown [Populus trichocarpa x Populus deltoides]	343	8.00E-93	1
CAO71287.1	unnamed protein product [Vitis vinifera]	343	8.00E-93	1
CAO14950.1	unnamed protein product [Vitis vinifera]	342	1.00E-92	1
ABK93552.1	unknown [Populus trichocarpa]	339	1.00E-91	1
CAO66650.1	unnamed protein product [Vitis vinifera]	336	1.00E-90	1
CAO42343.1	unnamed protein product [Vitis vinifera]	334	4.00E-90	1
CAO41275.1	unnamed protein product [Vitis vinifera]	334	5.00E-90	1
ABK96203.1	unknown [Populus trichocarpa]	331	3.00E-89	1
CAO66272.1	unnamed protein product [Vitis vinifera]	329	1.00E-88	1
CAO64218.1	unnamed protein product [Vitis vinifera]	256	2.00E-88	1
CAO48327.1	unnamed protein product [Vitis vinifera]	326	1.00E-87	1

ABK96305.1	unknown [Populus trichocarpa x Populus deltoides]	324	3.00E-87	1
ABK95775.1	unknown [Populus trichocarpa]	324	4.00E-87	1
CAN60740.1	hypothetical protein [Vitis vinifera]	323	6.00E-87	1
CAO18297.1	unnamed protein product [Vitis vinifera]	323	6.00E-87	1
CAO44273.1	unnamed protein product [Vitis vinifera]	321	4.00E-86	1
CAN80762.1	hypothetical protein [Vitis vinifera]	319	1.00E-85	1
CAO24389.1	unnamed protein product [Vitis vinifera]	318	2.00E-85	1
CAN75443.1	hypothetical protein [Vitis vinifera]	317	5.00E-85	1
CAO42285.1	unnamed protein product [Vitis vinifera]	315	3.00E-84	1
CAO22996.1	unnamed protein product [Vitis vinifera]	308	3.00E-82	1
BAF01964.1	hypothetical protein [Arabidopsis thaliana]	274	4.00E-82	3
CAO21314.1	unnamed protein product [Vitis vinifera]	305	2.00E-81	1
CAO40176.1	unnamed protein product [Vitis vinifera]	298	3.00E-79	1
CAO39385.1	unnamed protein product [Vitis vinifera]	297	4.00E-79	1
CAO67339.1	unnamed protein product [Vitis vinifera]	297	5.00E-79	1
CAO44301.1	unnamed protein product [Vitis vinifera]	297	5.00E-79	1
BAF75825.1	hypothetical protein [Malus x domestica]	297	5.00E-79	1
CAN64894.1	hypothetical protein [Vitis vinifera]	296	1.00E-78	1
CAO24542.1	unnamed protein product [Vitis vinifera]	294	4.00E-78	1
CAO66868.1	unnamed protein product [Vitis vinifera]	293	8.00E-78	1
CAO22937.1	unnamed protein product [Vitis vinifera]	292	2.00E-77	1
ABK94929.1	unknown [Populus trichocarpa]	288	2.00E-76	1
ABK92884.1	unknown [Populus trichocarpa]	288	2.00E-76	1
CAO40339.1	unnamed protein product [Vitis vinifera]	288	3.00E-76	1
CAO47376.1	unnamed protein product [Vitis vinifera]	287	4.00E-76	1
CAN72172.1	hypothetical protein [Vitis vinifera]	287	7.00E-76	1
CAO61102.1	unnamed protein product [Vitis vinifera]	286	8.00E-76	1
CAO62087.1	unnamed protein product [Vitis vinifera]	285	2.00E-75	1
CAO65647.1	unnamed protein product [Vitis vinifera]	285	2.00E-75	1
CAN64937.1	hypothetical protein [Vitis vinifera]	283	7.00E-75	1
CAO68379.1	unnamed protein product [Vitis vinifera]	280	9.00E-74	2
CAB51659.1	putative protein [Arabidopsis thaliana]	277	4.00E-73	1
ABK94241.1	unknown [Populus trichocarpa]	276	8.00E-73	1
CAO18146.1	unnamed protein product [Vitis vinifera]	275	1.00E-72	1
CAO16914.1	unnamed protein product [Vitis vinifera]	274	4.00E-72	1
CAO39662.1	unnamed protein product [Vitis vinifera]	274	5.00E-72	1
ABN05794.1	hypothetical protein MtrDRAFT_AC148817g33v2 [Medicago truncatula]	274	6.00E-72	1
CAO68979.1	unnamed protein product [Vitis vinifera]	273	8.00E-72	1
CAO21699.1	unnamed protein product [Vitis vinifera]	273	1.00E-71	2
ABK93406.1	unknown [Populus trichocarpa]	270	6.00E-71	1

AAM63369.1	unknown [Arabidopsis thaliana]	270	6.00E-71	1
CAN79693.1	hypothetical protein [Vitis vinifera]	268	2.00E-70	1
CAO66747.1	unnamed protein product [Vitis vinifera]	267	4.00E-70	1
CAO44335.1	unnamed protein product [Vitis vinifera]	257	5.00E-67	1
CAO16621.1	unnamed protein product [Vitis vinifera]	257	7.00E-67	1
CAO23276.1	unnamed protein product [Vitis vinifera]	256	9.00E-67	1
CAO63407.1	unnamed protein product [Vitis vinifera]	256	1.00E-66	1
CAO23149.1	unnamed protein product [Vitis vinifera]	256	1.00E-66	1
CAO40354.1	unnamed protein product [Vitis vinifera]	255	2.00E-66	1
CAO63610.1	unnamed protein product [Vitis vinifera]	256	2.00E-66	1
CAN80823.1	hypothetical protein [Vitis vinifera]	249	8.00E-65	1
CAO69187.1	unnamed protein product [Vitis vinifera]	247	6.00E-64	1
ABK95200.1	unknown [Populus trichocarpa]	247	7.00E-64	1
CAO14701.1	unnamed protein product [Vitis vinifera]	242	2.00E-62	1
CAO41446.1	unnamed protein product [Vitis vinifera]	242	2.00E-62	1
CAO22537.1	unnamed protein product [Vitis vinifera]	239	1.00E-61	1
CAO44173.1	unnamed protein product [Vitis vinifera]	239	2.00E-61	1
CAB95830.1	hypothetical protein [Cicer arietinum]	238	3.00E-61	1
ABK95057.1	unknown [Populus trichocarpa]	235	3.00E-60	1
ABK94930.1	unknown [Populus trichocarpa]	233	5.00E-60	1
ABK95546.1	unknown [Populus trichocarpa]	233	9.00E-60	1
CAO66902.1	unnamed protein product [Vitis vinifera]	233	1.00E-59	1
CAO65442.1	unnamed protein product [Vitis vinifera]	231	2.00E-59	1
CAO14711.1	unnamed protein product [Vitis vinifera]	229	1.00E-58	1
CAA18194.1	putative protein [Arabidopsis thaliana]	228	4.00E-58	1
ABK92614.1	unknown [Populus trichocarpa]	226	7.00E-58	1
CAO43759.1	unnamed protein product [Vitis vinifera]	225	2.00E-57	1
CAO71132.1	unnamed protein product [Vitis vinifera]	222	1.00E-56	1
CAO40896.1	unnamed protein product [Vitis vinifera]	221	3.00E-56	1
ABK93477.1	unknown [Populus trichocarpa]	220	5.00E-56	1
CAB37492.1	putative protein [Arabidopsis thaliana]	218	4.00E-55	1
CAO17698.1	unnamed protein product [Vitis vinifera]	217	6.00E-55	1
ABK94675.1	unknown [Populus trichocarpa]	216	1.00E-54	1
NP_190603.1	unknown protein [Arabidopsis thaliana]	215	2.00E-54	1
CAN62024.1	hypothetical protein [Vitis vinifera]	214	6.00E-54	1
CAO15843.1	unnamed protein product [Vitis vinifera]	212	2.00E-53	1
CAO49409.1	unnamed protein product [Vitis vinifera]	210	7.00E-53	1
CAN81044.1	hypothetical protein [Vitis vinifera]	209	7.00E-53	1
CAN65779.1	hypothetical protein [Vitis vinifera]	207	3.00E-52	1
CAN61153.1	hypothetical protein [Vitis vinifera]	208	3.00E-52	1

CAO64505.1	unnamed protein product [Vitis vinifera]	207	6.00E-52	1
CAO21400.1	unnamed protein product [Vitis vinifera]	207	6.00E-52	1
CAO68222.1	unnamed protein product [Vitis vinifera]	204	2.00E-51	1
CAI84658.1	hypothetical protein [Nicotiana tabacum]	205	2.00E-51	1
CAO45214.1	unnamed protein product [Vitis vinifera]	204	4.00E-51	1
CAO39210.1	unnamed protein product [Vitis vinifera]	203	1.00E-50	1
CAO16473.1	unnamed protein product [Vitis vinifera]	201	1.00E-50	1
CAN67814.1	hypothetical protein [Vitis vinifera]	202	2.00E-50	2
CAO70908.1	unnamed protein product [Vitis vinifera]	201	4.00E-50	1
CAN77019.1	hypothetical protein [Vitis vinifera]	158	5.00E-50	1
CAO61780.1	unnamed protein product [Vitis vinifera]	200	6.00E-50	1
ABK94537.1	unknown [Populus trichocarpa]	198	3.00E-49	1
ABK95430.1	unknown [Populus trichocarpa]	196	6.00E-49	1
ABK95743.1	unknown [Populus trichocarpa]	197	6.00E-49	1
CAO15686.1	unnamed protein product [Vitis vinifera]	196	8.00E-49	1
CAO70074.1	unnamed protein product [Vitis vinifera]	196	1.00E-48	1
CAN64895.1	hypothetical protein [Vitis vinifera]	194	3.00E-48	1
CAO61631.1	unnamed protein product [Vitis vinifera]	193	4.00E-48	1
CAO47802.1	unnamed protein product [Vitis vinifera]	194	4.00E-48	1
ABK94218.1	unknown [Populus trichocarpa]	187	6.00E-46	1
CAN65949.1	hypothetical protein [Vitis vinifera]	187	1.00E-45	1
CAO61623.1	unnamed protein product [Vitis vinifera]	184	3.00E-45	1
ABK93574.1	unknown [Populus trichocarpa]	185	4.00E-45	1
CAN71308.1	hypothetical protein [Vitis vinifera]	183	8.00E-45	1
ABK96152.1	unknown [Populus trichocarpa]	181	4.00E-44	1
CAN63850.1	hypothetical protein [Vitis vinifera]	181	4.00E-44	1
CAO21917.1	unnamed protein product [Vitis vinifera]	181	5.00E-44	1
CAO67077.1	unnamed protein product [Vitis vinifera]	181	6.00E-44	1
CAN80873.1	hypothetical protein [Vitis vinifera]	179	1.00E-43	1
CAO44961.1	unnamed protein product [Vitis vinifera]	179	1.00E-43	1
ABK93457.1	unknown [Populus trichocarpa]	178	2.00E-43	1
CAO38954.1	unnamed protein product [Vitis vinifera]	176	9.00E-43	1
ABK94521.1	unknown [Populus trichocarpa]	175	2.00E-42	1
CAO45481.1	unnamed protein product [Vitis vinifera]	174	3.00E-42	1
ABK93219.1	unknown [Populus trichocarpa]	170	5.00E-41	1
CAN64864.1	hypothetical protein [Vitis vinifera]	123	3.00E-40	1
CAO16953.1	unnamed protein product [Vitis vinifera]	167	4.00E-40	1
CAO43985.1	unnamed protein product [Vitis vinifera]	166	5.00E-40	1
ABK93697.1	unknown [Populus trichocarpa]	167	6.00E-40	1
CAO45573.1	unnamed protein product [Vitis vinifera]	149	9.00E-39	1

ABK94884.1	unknown [Populus trichocarpa]	161	4.00E-38	1
ABK22743.1	unknown [Picea sitchensis]	160	1.00E-37	1
CAN66505.1	hypothetical protein [Vitis vinifera]	159	1.00E-37	1
ABK94836.1	unknown [Populus trichocarpa]	158	3.00E-37	1
YP_588403.1	hypothetical protein ZeamMp158 [Zea mays subsp. mays]	151	3.00E-37	1
CAO41469.1	unnamed protein product [Vitis vinifera]	157	4.00E-37	1
ABD28368.1	hypothetical protein MtrDRAFT_AC148289g12v2 [Medicago truncatula]	156	7.00E-37	1
CAO71550.1	unnamed protein product [Vitis vinifera]	156	1.00E-36	1
CAO45477.1	unnamed protein product [Vitis vinifera]	154	2.00E-36	1
CAO44286.1	unnamed protein product [Vitis vinifera]	155	2.00E-36	1
CAO15629.1	unnamed protein product [Vitis vinifera]	99.4	2.00E-36	1
CAO48579.1	unnamed protein product [Vitis vinifera]	152	1.00E-35	1
CAB96688.1	putative protein [Arabidopsis thaliana]	152	2.00E-35	1
CAO15090.1	unnamed protein product [Vitis vinifera]	152	2.00E-35	1
CAO42363.1	unnamed protein product [Vitis vinifera]	152	2.00E-35	1
ABK93674.1	unknown [Populus trichocarpa]	151	3.00E-35	1
ABK94193.1	unknown [Populus trichocarpa]	151	3.00E-35	1
CAN61119.1	hypothetical protein [Vitis vinifera]	130	3.00E-35	1
CAO68283.1	unnamed protein product [Vitis vinifera]	151	4.00E-35	1
CAN74819.1	hypothetical protein [Vitis vinifera]	151	5.00E-35	1
CAO15842.1	unnamed protein product [Vitis vinifera]	145	2.00E-33	1
CAN62217.1	hypothetical protein [Vitis vinifera]	144	2.00E-33	1
ABK96304.1	unknown [Populus trichocarpa x Populus deltoides]	138	1.00E-31	1
CAO23602.1	unnamed protein product [Vitis vinifera]	138	4.00E-31	1
ABK94210.1	unknown [Populus trichocarpa]	135	2.00E-30	1
CAO16712.1	unnamed protein product [Vitis vinifera]	135	3.00E-30	1
CAO42932.1	unnamed protein product [Vitis vinifera]	134	4.00E-30	1
CAO69966.1	unnamed protein product [Vitis vinifera]	134	5.00E-30	1
CAO64960.1	unnamed protein product [Vitis vinifera]	132	6.00E-30	1
ABK92973.1	unknown [Populus trichocarpa]	131	2.00E-29	1
CAN79760.1	hypothetical protein [Vitis vinifera]	131	6.00E-29	1
CAO17337.1	unnamed protein product [Vitis vinifera]	130	1.00E-28	1
CAO67112.1	unnamed protein product [Vitis vinifera]	128	3.00E-28	1
ABK93131.1	unknown [Populus trichocarpa]	127	4.00E-28	1
CAN62511.1	hypothetical protein [Vitis vinifera]	124	7.00E-27	1
CAO50031.1	unnamed protein product [Vitis vinifera]	124	8.00E-27	1
CAO66422.1	unnamed protein product [Vitis vinifera]	121	2.00E-26	1
CAO70557.1	unnamed protein product [Vitis vinifera]	120	8.00E-26	1
ABK92578.1	unknown [Populus trichocarpa]	118	1.00E-25	1
ABK94311.1	unknown [Populus trichocarpa]	117	2.00E-25	1

CAA10123.1	hypothetical protein [Cicer arietinum]	118	4.00E-25	1
ABK96690.1	unknown [Populus trichocarpa x Populus deltoides]	115	2.00E-24	1
CAO43566.1	unnamed protein product [Vitis vinifera]	115	4.00E-24	1
CAO24691.1	unnamed protein product [Vitis vinifera]	112	1.00E-23	1
CAO14801.1	unnamed protein product [Vitis vinifera]	107	2.00E-23	1
ABK96142.1	unknown [Populus trichocarpa]	112	2.00E-23	1
ABK93587.1	unknown [Populus trichocarpa]	110	4.00E-23	1
CAO70312.1	unnamed protein product [Vitis vinifera]	110	4.00E-23	1
CAB77598.1	putative protein [Arabidopsis thaliana]	108	2.00E-22	1
CAO67231.1	unnamed protein product [Vitis vinifera]	102	8.00E-21	1
CAO45301.1	unnamed protein product [Vitis vinifera]	102	9.00E-21	1
CAO68988.1	unnamed protein product [Vitis vinifera]	103	1.00E-20	1
CAO71442.1	unnamed protein product [Vitis vinifera]	102	3.00E-20	1
CAO43782.1	unnamed protein product [Vitis vinifera]	101	4.00E-20	1
NP_191358.1	unknown protein [Arabidopsis thaliana]	99.4	2.00E-19	1
ABK93164.1	unknown [Populus trichocarpa]	98.2	2.00E-19	1
CAN77855.1	hypothetical protein [Vitis vinifera]	98.6	4.00E-19	1
CAN82375.1	hypothetical protein [Vitis vinifera]	98.2	4.00E-19	1
CAN82126.1	hypothetical protein [Vitis vinifera]	95.1	1.00E-18	1
CAN70790.1	hypothetical protein [Vitis vinifera]	97.1	1.00E-18	1
AAM61659.1	unknown [Arabidopsis thaliana]	92	1.00E-17	1
CAO16187.1	unnamed protein product [Vitis vinifera]	90.9	2.00E-17	1
CAO42580.1	unnamed protein product [Vitis vinifera]	90.9	8.00E-17	1
ABK95674.1	unknown [Populus trichocarpa]	88.2	2.00E-16	1
CAO71849.1	unnamed protein product [Vitis vinifera]	89.7	2.00E-16	1
ABK93149.1	unknown [Populus trichocarpa]	88.2	3.00E-16	1
CAO14772.1	unnamed protein product [Vitis vinifera]	88.6	4.00E-16	1
CAO40090.1	unnamed protein product [Vitis vinifera]	88.2	6.00E-16	1
ZP_00874766.1	hypothetical protein SsuiDRAFT_1635 [Streptococcus suis 89/1591]	67.8	3.00E-15	1
CAO38914.1	unnamed protein product [Vitis vinifera]	82	1.00E-14	1
P26563|AATM_LUPAN	Aspartate aminotransferase P2, mitochondrial precursor (Transaminase A)	80.9	3.00E-14	1
CAO65372.1	unnamed protein product [Vitis vinifera]	80.9	3.00E-14	1
CAO47252.1	unnamed protein product [Vitis vinifera]	79.3	9.00E-14	1
CAO67977.1	unnamed protein product [Vitis vinifera]	78.2	3.00E-13	1
ABK23797.1	unknown [Picea sitchensis]	74.3	4.00E-12	1
CAO40846.1	unnamed protein product [Vitis vinifera]	59.7	8.00E-12	1
ABK93285.1	unknown [Populus trichocarpa]	72	1.00E-11	1
CAO45180.1	unnamed protein product [Vitis vinifera]	72.4	2.00E-11	1
CAO48073.1	unnamed protein product [Vitis vinifera]	70.9	6.00E-11	1
XP_001067213.1	PREDICTED: hypothetical protein [Rattus norvegicus]	68.9	4.00E-10	1

CAO68098.1	unnamed protein product [Vitis vinifera]	497	1.00E-139	1
CAO21841.1	unnamed protein product [Vitis vinifera]	479	1.00E-134	1
CAO23214.1	unnamed protein product [Vitis vinifera]	429	1.00E-118	1
ABK93489.1	unknown [Populus trichocarpa]	407	1.00E-112	1
CAO63600.1	unnamed protein product [Vitis vinifera]	397	1.00E-109	1
ABK93226.1	unknown [Populus trichocarpa]	378	1.00E-103	1
CAO63606.1	unnamed protein product [Vitis vinifera]	377	1.00E-103	1
CAO16101.1	unnamed protein product [Vitis vinifera]	372	1.00E-101	1
CAO69484.1	unnamed protein product [Vitis vinifera]	342	2.00E-92	1
NP_171983.2	unknown protein [Arabidopsis thaliana]	336	1.00E-90	1
ABK96759.1	unknown [Populus trichocarpa x Populus deltoides]	322	2.00E-86	1
CAN81221.1	hypothetical protein [Vitis vinifera]	316	1.00E-84	1
ABA46755.1	unknown [Solanum tuberosum]	292	2.00E-77	1
NP_568958.1	unknown protein [Arabidopsis thaliana]	159	5.00E-76	1
ABK93056.1	unknown [Populus trichocarpa]	219	1.00E-55	2
ABK96157.1	unknown [Populus trichocarpa]	202	6.00E-51	1
CAO42313.1	unnamed protein product [Vitis vinifera]	199	1.00E-49	1
ABK96772.1	unknown [Populus trichocarpa x Populus deltoides]	197	5.00E-49	1
CAO24297.1	unnamed protein product [Vitis vinifera]	151	3.00E-35	1
CAO14817.1	unnamed protein product [Vitis vinifera]	151	5.00E-35	1
CAO16964.1	unnamed protein product [Vitis vinifera]	150	8.00E-35	1
XP_001269594.1	hypothetical protein ACLA_028940 [Aspergillus clavatus NRRL 1]	73.6	1.00E-22	2
CAO41541.1	unnamed protein product [Vitis vinifera]	108	2.00E-22	1
NP_565728.1	unknown protein [Arabidopsis thaliana]	106	1.00E-21	1
EDP38581.1	hypothetical protein Bm1_05555 [Brugia malayi]	92	4.00E-17	1
ABK94233.1	unknown [Populus trichocarpa]	62	1.00E-08	1
ABN08062.1	Protein of unknown function DUF632 [Medicago truncatula]	52.8	1.00E-05	1
CAO40971.1	unnamed protein product [Vitis vinifera]	129	2.00E-28	1
CAO22611.1	unnamed protein product [Vitis vinifera]	183	9.00E-45	1
